# From Principles to Practice: Engineering Responsible AI for Geospatial Intelligence

**DOI:** 10.1007/s42979-026-05258-0

**Published:** 2026-08-13

**Authors:** Muhammad Hassan, Bilal Sardar, Shareeful Islam, Maryam Imani, Akram Hakiri

**Affiliations:** 1 School of Computing and Information Science, Anglia Ruskin University, East Road, Cambridge, UK; 2 School of Engineering and the Built Environment, Anglia Ruskin University, Chelmsford, UK; 3 Department of Computer Science, Université de Pau et des Pays de l’Adour (UPPA), 2 Allée du Parc Montaury, Anglet, 64600 Nouvelle-Aquitaine France

**Keywords:** Responsible AI, Geospatial AI, Multi-Temporal Satellite Classification, Explainable AI, Temporal Attribution

## Abstract

AI-enabled geospatial applications increasingly inform high-stakes decisions in crop type classification, flood risk assessment, and land use monitoring; yet current practice prioritises predictive accuracy whilst leaving responsible AI (R-AI) largely unaddressed. This work aims to operationalise R-AI practice for geospatial AI by proposing a conceptual view and a four-phase methodology that embeds four distinct R-AI characteristics into the model development lifecycle. A conceptual R-AI view is proposed that situates four characteristics - Privacy, Fairness, Transparency, and Explainability - across three operational levels: data, model, and prediction. A four-phase methodology operationalises each through concrete techniques and quantifiable acceptance criteria. The threshold values and phase ordering of the methodology are governed by the proposed Pareto-Adaptive R-AI Compliance (PARC) algorithm, which derives dataset-specific thresholds and characterises the interactions between the four characteristics. Evaluation is conducted through two experiments using the Temporal-Spatial Vision Transformer (TSViT): crop type classification on the publicly available PASTIS/EuroCrops benchmark, and urban flood risk classification on a real-world UK Sustainable Drainage Systems (SuDS) pilot with six fused geospatial data sources. Across both experiments, all twelve R-AI acceptance criteria are satisfied. Privacy protection reduces membership inference attack accuracy from 0.712 to 0.503, approaching the random-guess baseline. Fairness interventions narrow the geographic performance gap from 23.1% to 3.1%. Transparency verification reduces physically inconsistent model behaviour from 24.1% to 3.8%. Explainability mechanisms improve temporal attribution consistency from 0.41 to 0.857, and the attribution methods are independently validated through deletion, insertion, and perturbation-stability analyses. All four characteristics are achieved whilst retaining over 97% of baseline predictive utility. These findings demonstrate that responsible AI and predictive accuracy are compatible objectives in geospatial deep learning. The proposed approach offers a replicable and measurable pathway for embedding R-AI practice into geospatial AI development, validated across two structurally distinct real-world tasks.

## Introduction

Artificial Intelligence (AI) has become a core enabler of modern Geographic Information Systems (GIS), fundamentally transforming how geospatial data are analysed and applied. Powered by openly accessible satellite data - most notably the Sentinel-2 time series, which delivers dense, 10-metre resolution multi-temporal imagery across the entire Earth surface - AI-enabled GIS systems now support applications ranging from crop type mapping and flood risk assessment to land use monitoring and environmental analysis at scales that were simply not achievable a decade ago [1–3]. Consequently, the outputs of these systems are increasingly used to inform high-stakes decisions in agricultural monitoring, infrastructure planning, and environmental reporting [3, 4]. This growing reliance, however, brings an important responsibility: geospatial AI systems must not only be accurate, but also handle sensitive data responsibly, treat different populations equitably, and produce decisions that domain experts can understand and trust.

In practice, these requirements are often unmet. A striking example emerged from crop type mapping efforts across sub-Saharan Africa, where AI models trained predominantly on satellite data from Western agricultural contexts were found unable to recognise common local crops such as maize, beans, and cassava in Kenya, rendering the systems effectively useless for the smallholder farmers they were designed to serve [5, 6]. The failure was not architectural; it was a failure of responsible practice - training data that did not represent the target population, with no mechanism to detect or correct that gap before deployment. Related concerns have since been documented closer to the geospatial domains of this work: geographic bias in geospatial datasets has been shown to produce consistently lower reliability for underrepresented regions than for well-represented areas [7], whilst trained remote sensing models have been found to leak sensitive land parcel records through membership inference attacks, exposing ownership and subsidy information without the knowledge of affected parties [8]. These failures share a common root: geospatial AI development has prioritised predictive accuracy whilst leaving the responsible dimensions of model behaviour largely unaddressed.

Several recent studies have begun to address parts of this gap, yet a unified solution has yet to emerge. Tarasiou et al. [2] advanced multi-temporal satellite classification through the Temporal-Spatial Vision Transformer (TSViT), yet their work focused exclusively on predictive performance. Gevaert et al. [7] identified dataset bias in geospatial applications, and Keßler and McKenzie [8] characterised privacy risks from spatial quasi-identifiers in land parcel data - yet neither offers an integrated solution that embeds mitigation into the development process itself. What the field therefore still lacks is a unified approach that addresses the full scope of responsible AI practice, maps each concern to a concrete stage of the AI development lifecycle, and validates the outcome through measurable criteria on real geospatial tasks.

To address this, the present paper proposes a conceptual Responsible AI (R-AI) view for geospatial intelligence that identifies four distinct characteristics - Privacy, Fairness, Transparency, and Explainability - each situated at a specific operational level of the AI development lifecycle. Building on this view, a four-phase methodology is proposed that systematically embeds all four characteristics into model development, with each phase governed by quantifiable acceptance criteria. The approach is validated through two structurally distinct experiments: crop type classification using publicly available multi-temporal satellite data, and urban flood risk classification on a real-world UK geospatial pilot. The contributions of this paper are as follows.

- Firstly, we propose a conceptual R-AI view that embeds four characteristics - Privacy, Fairness, Transparency, and Explainability - each at a specific operational level: Privacy at the data level, protecting sensitive land parcel records through geo-indistinguishability and spatial k-anonymity; Fairness at the model level, promoting geographic equity through fairness-aware training; and Transparency and Explainability through evaluation and deployment, enforcing physical consistency via metamorphic testing and linking model attention to agronomically validated events through spatial, spectral, and temporal attribution - including the novel Temporal Attribution Consistency (TAC) metric.
- Secondly, beyond the system-level integration of established techniques, we contribute the Pareto-Adaptive R-AI Compliance (PARC) algorithm, which transforms the methodology from a fixed pipeline with assumed thresholds into a data-driven procedure. PARC derives each acceptance threshold from a domain-meaningful reference distribution on the target dataset, constructs an R-AI Interdependency Matrix that makes the trade-offs between the four characteristics empirically measurable, adaptively orders the four phases to minimise destructive interference, and selects the configuration that maximises the minimum normalised slack across all criteria. The matrix, the adaptive ordering, and the resulting slack certificate are elements that prior R-AI pipelines do not provide.
- Thirdly, we present a methodology that operationalises all four R-AI characteristics across four sequential development phases. Phase 1 (Data Collection and Preprocessing) applies privacy protections before any model training, safeguarding sensitive records from the outset. Phase 2 (Model Training and Development) embeds geographic fairness constraints into the optimisation objective, ensuring equitable learning across all regions [9]. Phase 3 (Model Evaluation and Transparency Verification) tests predictions against physics-grounded behavioural rules and produces structured documentation for independent audit. Phase 4 (Deployment and Explainability) delivers spatial attribution via GradCAM++, spectral attribution via Integrated Gradients, and temporal attribution via TAC, providing a complete and domain-interpretable account of each prediction. Each phase is governed by quantifiable acceptance criteria, enforcing R-AI compliance as measurable engineering constraints.
- Finally, we validate the approach through two structurally distinct experiments using TSViT as the shared model backbone. Experiment 1 covers crop type classification on the publicly available PASTIS benchmark with EuroCrops labels across nine crop classes. Experiment 2 applies the same approach to a real-world UK Sustainable Drainage Systems (SuDS) flood risk classification pilot, involving six heterogeneous geospatial data sources and five ordinal risk levels. In Experiment 1, privacy protection reduces membership inference attack accuracy from 71% to near-random (50%); fairness interventions narrow the geographic performance gap from 23.1% to 3.1%; transparency verification reduces physically inconsistent model behaviour from 24.1% to 3.8%; and explainability mechanisms confirm that model attention aligns with validated seasonal events rather than data artefacts - all whilst retaining over 97% of baseline predictive utility. In Experiment 2, the same methodology reduces MIA accuracy from 69.4% to 51.1%, narrows the geographic performance gap from 14.9% to 4.8%, reduces metamorphic testing violations from 21.8% to 5.2%, and achieves temporal attribution consistency of 0.82 - whilst retaining over 98% of baseline predictive utility. Across both experiments, all twelve R-AI acceptance criteria are satisfied, demonstrating that the proposed approach generalises across structurally distinct geospatial tasks. The validation is further deepened through significance testing and confidence intervals over five seeds, a leave-one-phase-out ablation establishing the non-redundancy of each characteristic, a comparison against single-characteristic privacy-only, fairness-only, and explainability-only pipelines, and a faithfulness, perturbation-stability, and expert-assessment validation of the attribution methods.

## Related Work

### Responsible AI: Principles and Frameworks

Responsible AI (R-AI) refers to the design and deployment of AI systems that are fair, transparent, explainable, and privacy-preserving throughout their full lifecycle. These four principles are not independent add-ons but are deeply intertwined: a model that maximises accuracy may simultaneously be unfair to minority groups, while a fully transparent model may expose sensitive personal data [8, 10]. To address this, Barletta et al. [11] conducted a rapid review of existing R-AI frameworks and found that most provide guidelines only at the requirements stage, leaving the critical phases of model training, validation, and post-deployment monitoring without responsible governance. This means that even well-intentioned R-AI efforts often fail at the point where harm is most likely to occur - during and after model development.

Addressing this lifecycle gap, Haidar [12] proposed an integrative theoretical framework that reasons explicitly about conflicting objectives at every design decision point. For instance, in a crop classification pipeline, optimising model accuracy for dominant crop types can reduce fairness for rare or minority crop classes-a tension that must be resolved deliberately rather than left to emerge at deployment. Furthermore, this requires R-AI to be treated not as a technical property alone, but as an organisational commitment spanning technical compliance and legal alignment. At the regulatory level, the EU AI Act [13] formalised these obligations for high-risk AI systems, requiring transparency and bias mitigation; however, Walke et al. [14] noted that the Act’s explainability requirements remain abstract and difficult to operationalise for specific AI applications. Taken together, these works establish that R-AI is a governance challenge as much as a technical one - and that embedding it into an AI pipeline from the outset is essential.

### Responsible AI in GIS

Applying R-AI principles in geospatial AI introduces challenges that general-purpose frameworks are not designed to handle. Marasinghe et al. [4] reviewed responsible practices for urban GeoAI and found that most existing R-AI literature addresses general principles without offering domain-specific guidance for spatial contexts. They showed that fairness, explainability, and transparency are especially critical in GeoAI because spatial AI outputs - such as crop maps or flood risk zones - directly inform high-stakes decisions about land use, food security, and environmental policy. Building on this, Ghamisi et al. [3] argued specifically in the context of Earth observation that responsible AI must be systematically embedded into remote sensing pipelines, not treated as an optional enhancement. They highlighted that errors in AI-driven Earth observation - such as misclassifying crop types at regional scale - can have significant downstream consequences for agricultural monitoring and resource allocation.

Fairness in GeoAI requires spatial metrics that go beyond standard demographic parity. Saxena et al. [15] formalised the need for spatial fairness, showing that geographic location introduces biases that conventional metrics cannot detect - for example, when training data is densely sampled in irrigated lowland fields but sparse in upland or marginal agricultural zones. Gevaert et al. [7] confirmed this empirically, demonstrating that spatial coverage biases in geospatial datasets directly degrade model reliability in underrepresented areas. On explainability, Xing and Sieber [16] identified that standard attribution methods such as Grad-CAM and SHAP fail to respect spatial autocorrelation and geographic scale when applied without spatial adaptation, arguing that GeoXAI must provide explanations meaningful to domain experts rather than purely computational attribution maps. These spatial fairness and explainability challenges are directly relevant to crop type prediction, where models must generalise reliably across varied agricultural landscapes and provide interpretable outputs that agronomists and policymakers can trust and act upon.

The stage at which fairness is enforced is itself a design decision. Ntoutsi et al. [17] provide a fish-eye survey of bias mitigation in algorithmic systems and distinguish three intervention points: pre-processing, which rebalances or repairs the training data; in-processing, which constrains the learning objective; and post-processing, which adjusts the model outputs after training. Each has a characteristic strength: pre-processing acts before any model is fitted, in-processing can shape the decision boundary directly, and post-processing requires no retraining. In the geospatial setting of this work, fairness is enforced primarily as an in-processing intervention because the source of geographic disadvantage is representational - benchmark datasets such as EuroCrops systematically underrepresent certain regions - and information that the model never learns equitably cannot be reliably recovered by adjusting outputs after the fact [15]. Pre-processing is nonetheless partially present in the proposed approach through stratified geographic sampling, which rebalances each training batch, while output-stage post-processing is deliberately avoided because per-region threshold adjustment on flood-risk or subsidy-linked predictions is difficult to justify to the affected stakeholders. The complementary value of stronger pre- and post-processing interventions is revisited in the discussion as a direction for future work.

### Transformer-Based Crop Type Classification from Satellite Imagery

Crop type classification from satellite imagery has advanced significantly with the adoption of transformer-based architectures, which are well-suited to the temporal and spectral complexity of agricultural remote sensing data. Unlike convolutional neural networks, transformers use self-attention mechanisms to model long-range dependencies across spectral bands and time steps simultaneously, making them particularly effective for Sentinel-2 multispectral time-series data where the spectral signature of a crop evolves across a growing season [2]. Rußwurm and Körner [18] demonstrated that temporal self-attention models outperform recurrent architectures for crop classification, as they can selectively attend to the most discriminative phenological stages - such as peak greenness or harvest - without being constrained by sequential processing order. This flexibility is critical in agricultural settings where cloud cover and irregular revisit intervals create gaps in the time-series that sequential models struggle to handle.

However, the adoption of transformers for crop classification also raises important responsible AI concerns that are not yet well addressed in the literature. The self-attention mechanism, while powerful, produces explanations that are difficult to interpret: standard attention maps show where the model looked, but not why a particular spectral-temporal pattern was decisive for predicting a specific crop type. Methods such as Attention Rollout and the Chefer method [19, 20] have been proposed to provide more faithful class-specific attribution for Vision Transformers, yet these techniques have seen limited application in agricultural remote sensing. Furthermore, transformer models trained on data from well-sampled agricultural regions may perform poorly and produce unfair predictions for underrepresented crop types or geographically marginal fields, a fairness concern that is directly linked to training data coverage and rarely audited in existing crop classification pipelines. These gaps motivate the integration of an explicit R-AI approach into the transformer-based crop classification pipeline developed in this work.

In summary, the existing literature remains critically fragmented - treating privacy, fairness, transparency, and explainability as isolated concerns rather than interdependent, lifecycle-spanning constraints. General R-AI frameworks stall at requirements elicitation without operationalising principles into actionable steps, GeoAI research identifies spatial challenges without concrete interventions, and transformer-based classification systems optimise for predictive performance while bypassing responsible AI auditing entirely. Critically, no existing work embeds these four characteristics as measurable, systematically enforced constraints within a unified operational framework - a gap especially consequential where outputs directly shape agricultural policy and resource allocation, demanding systems that are verifiably safe, fair, and ethically accountable.

## Conceptual View of Responsible AI

Responsible AI (R-AI) encompasses the design, development, and deployment of AI systems that prioritise human welfare, equitable treatment, and societal accountability throughout the AI lifecycle. Its adoption is particularly critical in GIS applications, where geospatial data introduces quasi-identification risks, geographic representation bias, and interpretability demands that make R-AI practice not merely desirable but necessary. To address these challenges, the proposed R-AI view organises four distinct characteristics - Privacy, Fairness, Transparency, and Explainability - across two fundamental dimensions of responsibility: Ethical Accountability and Interpretive Transparency, spanning three operational levels of the AI development lifecycle:

- Data level, which concerns the collection, preprocessing, and protection of training data before any model is built;
- Model level, which addresses the design, training, and internal behaviour of the AI system;
- Prediction level, which governs the outputs the model produces and how they are communicated to end users.

At the data level, privacy safeguards protect sensitive geospatial information before any model encounters the training set; at the model level, fairness and transparency are embedded into the learning process to ensure equitable and auditable decision-making; and at the prediction level, explainability enables domain experts to interrogate and validate the model’s outputs in operational contexts.

### Privacy

Privacy preservation refers to the protection of sensitive, spatially identifiable information from being leaked or inferred through AI model outputs [8]. In geospatial intelligence, land parcel boundaries, crop type declarations, and precise location coordinates are directly linked to individual landowners and farmers, constituting personal data whose exposure carries legal and financial consequences. A geospatial AI model trained on such data can inadvertently encode individual records in its weights, making it possible for adversaries to reconstruct sensitive attributes - such as field ownership, subsidy eligibility, or property flood risk grading - through model queries alone. Privacy preservation therefore applies protective mechanisms at the data and training levels to ensure that model outputs remain statistically indistinguishable whether or not any individual record was included in training.

- **Membership Inference Attack (MIA)** [21] measures whether adversaries can determine if a specific land parcel was used in model training by analysing prediction confidence patterns: Attack Accuracy = Correct Membership Predictions / Total Predictions, with lower rates indicating stronger protection.
- **Attribute Inference Attack (AIA)** [8] assesses how accurately adversaries can infer sensitive geospatial attributes - such as crop type or parcel ownership - that were excluded from model inputs, revealing whether the model has learned implicit correlations between observable spectral features and protected land-use attributes.
- **Distance to Closest Record (DCR)** [8] quantifies re-identification risk by computing the minimum spatial distance between synthetic and real records, $\text {DCR} = \min d(r,s)$ for *r ∈ R*, *s ∈ S*. Larger distances provide stronger guarantees that synthetic parcels cannot be traced back to actual farmers.
- **Differential Privacy** $(\varepsilon , \delta )$ [22] provides the formal guarantee
  1 $$\begin{aligned} P[M(D_{1}) \in S] \le e^{\varepsilon } \cdot P[M(D_{2}) \in S] + \delta , \end{aligned}$$
  where $D_1$ and $D_2$ differ by a single parcel record, *\varepsilon * is the privacy budget (smaller is stronger), and *δ * is the failure probability.

### Fairness

Fairness in AI requires that all subgroups receive equitable treatment from a model, without systematic disadvantage arising from attributes such as geographic location or group membership [23]. Publicly available satellite benchmark datasets are geographically skewed: EuroCrops over-represents Western European large-scale commercial farms whilst under-representing Eastern European smallholder systems, causing models to perform significantly worse for the very subgroups most dependent on accurate classification for subsidy access [6]. Geographic fairness therefore requires that equity constraints be embedded directly into the model training objective, rather than applied as post-hoc corrections [24].

- **Equal Opportunity** [23] requires equal True Positive Rates across subgroups, $TPR_{\text {small}} = TPR_{\text {major}}$, ensuring merit-based outcomes regardless of regional representation.
- **Demographic Parity** [23] requires equal positive-prediction rates across subgroups,
  2 $$\begin{aligned} P({\hat{Y}} = 1 \mid A = \text {minority}) = P({\hat{Y}} = 1 \mid A = \text {majority}), \end{aligned}$$
  preventing systematic exclusion of any region from beneficial outputs.
- **Equalized Odds** [23] extends Equal Opportunity to require both $TPR_{\text {small}} = TPR_{\text {major}}$ and $FPR_{\text {small}} = FPR_{\text {major}}$, preventing imbalanced error rates of either type.

### Transparency

Transparency refers to the capacity to document and verify how an AI system functions, covering the data it was trained on, the algorithmic decisions made during development, and the conditions under which its outputs are reliable [13]. Beyond documentation, transparency in geospatial AI requires that model predictions respect the physical laws of the domain - for instance, that a parcel with sustained high vegetation index cannot be classified as bare soil. Both dimensions are measured through structured documentation checklists and domain-aware behavioural testing.

- **Model Documentation (Model Card)** [25] provides a structured record of data provenance, preprocessing, model architecture, training configuration, and known limitations. The Documentation Score is the proportion of required fields populated with substantive content.
- **Metamorphic Testing** encodes physical invariances of satellite imagery as metamorphic relations (MRs); the MR Violation Rate is the proportion of test cases where the model output violates a defined physical invariance.

### Explainability

Explainability refers to the capacity of an AI system to provide human-interpretable justifications for its decisions [26]. In geospatial classification, attribution methods designed for static images are insufficient because satellite data is inherently multi-dimensional: a correct explanation must identify which spatial region the model attended to, which spectral bands carried the most discriminative information, and which acquisition dates were most influential [27].

- **GradCAM++** [27] generates spatial attribution heatmaps,
  3 $$\begin{aligned} L^{c}_{\text {GradCAM++}} = \textrm{ReLU}\Big (\sum _{k} \alpha ^{c}_{k} \cdot A_{k}\Big ), \end{aligned}$$
  where $A_k$ is the *k*-th final convolutional feature map and $\alpha ^{c}_{k}$ is its second/third-order-gradient-weighted importance for class *c*.
- **Temporal Attention Visualisation** extracts attention weights assigned to each acquisition date, with temporal importance $I_t = \frac{1}{H}\sum _h \alpha ^{h}_{t}$ averaged across *H* attention heads.
- **Integrated Gradients** [28] attributes the prediction to individual spectral bands by path-integrating gradients between a baseline *x'* and the actual observation *x*, satisfying the completeness axiom:
  4 $$\begin{aligned} IG_{i}(x) = (x_{i} - x'_{i}) \int _{0}^{1} \frac{\partial F\bigl (x' + \alpha (x - x')\bigr )}{\partial x_{i}}\, d\alpha . \end{aligned}$$

**Fig. 1:**
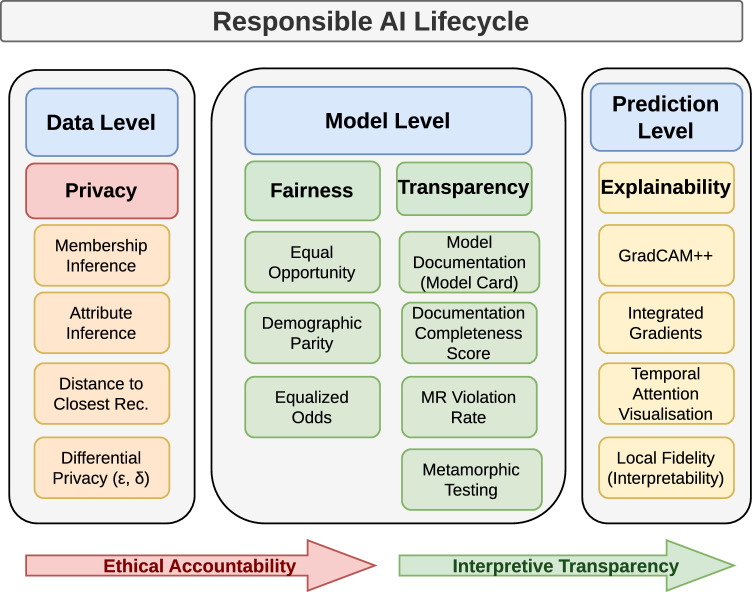
An Overview of Conceptual R-AI Lifecycle

Figure 1 presents a hierarchical view of the Responsible AI lifecycle, organising the four essential characteristics across three operational levels that address responsibility at different stages of development. At the Data Level, privacy protection employs four metrics to safeguard sensitive geospatial information before model training, establishing the foundational ethical layer. The Model Level encompasses fairness and transparency - fairness metrics address geographic and demographic bias across subgroups, while transparency mechanisms ensure auditable documentation and domain-aware verification of model behaviour. Finally, at the Prediction Level, explainability methods enable agronomists, land managers, and policy makers to understand and validate model decisions through geospatially-adapted attribution and interpretability techniques. This hierarchical organisation reflects a fundamental principle: responsibility must be systematically built across the entire AI lifecycle, where privacy-protected data enables fair model training, fair and transparent models support verifiable predictions, and verifiable predictions combined with explainability create deployable, trustworthy geospatial AI systems [10].

## Methodology

**Fig. 2:**
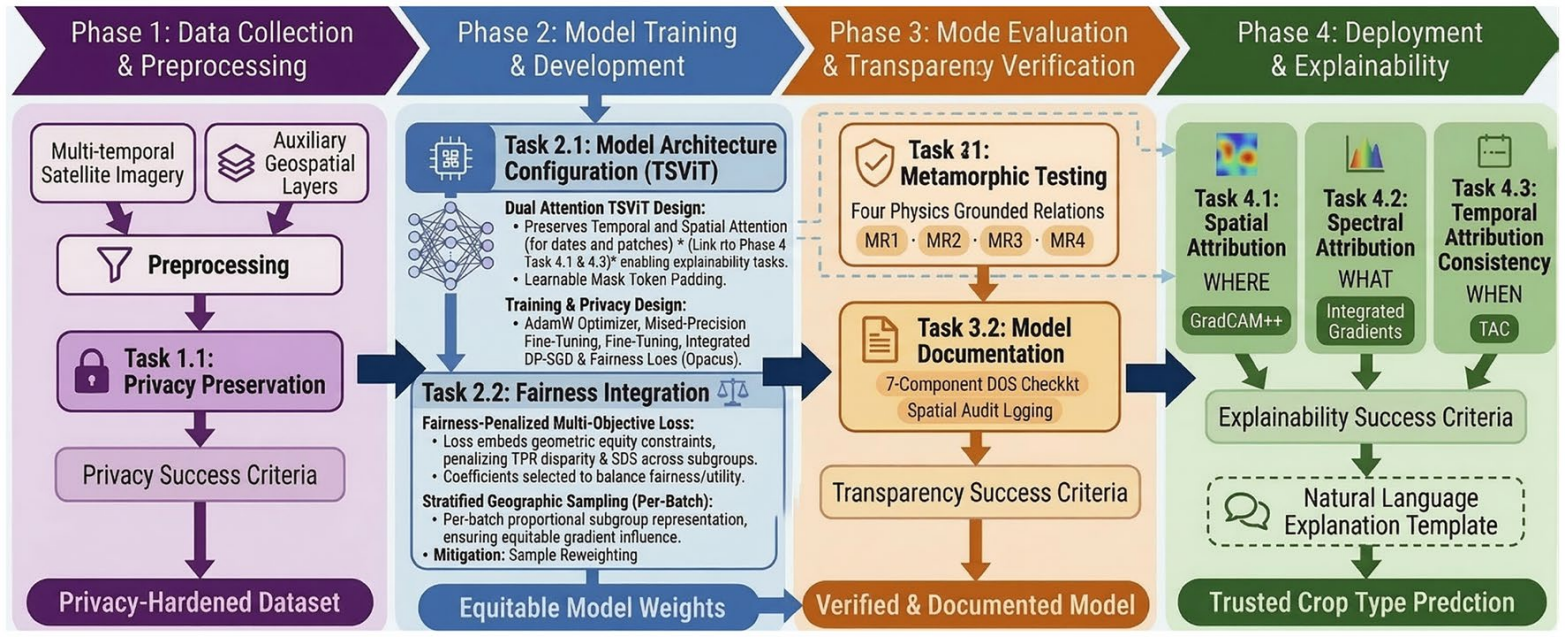
Methodology for Operationalise R-AI

This section outlines the proposed unified methodology that systematically integrates the two fundamental dimensions of responsible AI-Ethical Accountability and Interpretive Transparency-across the geospatial AI development lifecycle. As illustrated in Fig. 2, the proposed approach operationalises four key characteristics through four sequential phases, from data collection to deployment. The pipeline is designed to be model-agnostic; this work employs the Temporal-Spatial Vision Transformer (TSViT) [2] as the primary architecture to demonstrate applicability across multi-temporal satellite classification tasks. Unlike standard geospatial AI development pipelines that prioritise predictive accuracy above all else, this pipeline employs quantifiable success criteria for each R-AI characteristic, ensuring that responsible AI principles are not aspirational additions but measurable engineering constraints [10]. The phases are strictly sequential: each stage builds upon the previous one, ensuring all dimensions are integrated holistically rather than in isolation. The proposed approach maps the responsible AI dimensions to specific operational levels and development phases:

**Ethical Accountability:** This spans the Data Level (Phase 1) and Model Level (Phase 2) by embedding privacy protections and geographic fairness constraints into data preparation and model training respectively.

**Interpretive Transparency:** This is achieved at the Model Level (Phase 3) and Prediction Level (Phase 4) through domain-aware behavioural verification and geospatially adapted explainability mechanisms that enable regulators and domain experts to audit and validate model decisions.

Each of these phases, however, is governed by quantifiable thresholds whose values directly determine whether the approach succeeds on a given dataset, and the four characteristics interact in ways that a fixed sequential pipeline cannot capture - tightening privacy can degrade fairness, and tightening fairness can distort the attention patterns on which explainability depends. To address both concerns within a single principled procedure, the methodology is governed by the Pareto-Adaptive R-AI Compliance (PARC) algorithm, presented in Section Pareto-Adaptive R-AI Compliance (PARC) Algorithm, which derives dataset-appropriate thresholds and characterises these interactions before the four phases are executed.

### Pareto-Adaptive R-AI Compliance (PARC) Algorithm

The proposed approach integrates established techniques - differential privacy, fairness-aware optimisation, metamorphic testing, GradCAM++, and Integrated Gradients - yet its methodological contribution lies not in these components individually but in how they are jointly governed across the lifecycle. Two difficulties make this governance non-trivial: each of the twelve acceptance criteria is gated by a threshold whose appropriate value is dataset-dependent rather than universal, and the four characteristics are not independent - enforcing one can move another away from its threshold. PARC, summarised in Algorithm 1 and executed once on the target dataset before the four phases begin, resolves both difficulties in four steps. *Step 0* anchors each of the twelve thresholds to a reference distribution computed on the target dataset, so every threshold carries a statistical grounding rather than a heuristic value (specific protocols and derived values in Table 1). *Step 1* uses a small Latin hypercube probe over the joint configuration space to fit Gaussian Process surrogates and compute the R-AI Interdependency Matrix *M*, in which $M_{ij}$ quantifies how tightening criterion *j* affects criterion *i*. *Step 2* uses *M* to reorder the four phases so as to minimise destructive interference between the most conflicting characteristics. *Step 3* selects, among all feasible configurations, the one that maximises the minimum normalised slack across all criteria, producing a slack certificate that records the margin by which each threshold is met.

**Algorithm 1:**
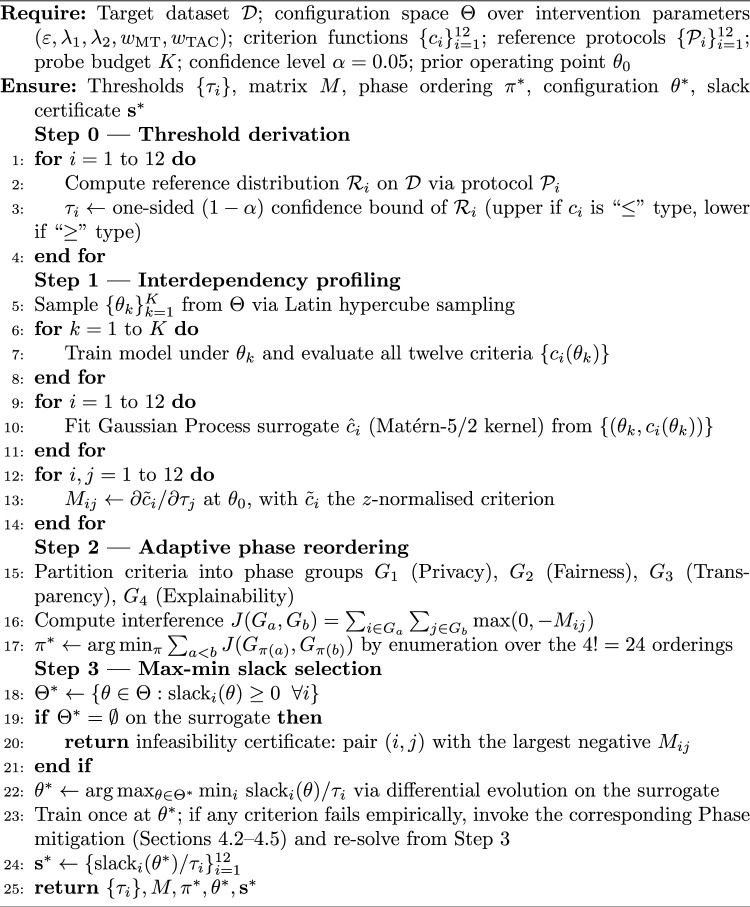
Pareto-Adaptive R-AI Compliance (PARC)

Together, these four steps contribute three elements that distinguish PARC from a system-level integration of existing techniques: the threshold derivation step removes the dependence on heuristic values, so the procedure transfers to a previously unseen geospatial dataset without carrying over assumptions; the Interdependency Matrix *M* makes trade-offs that were previously discussed only qualitatively empirically measurable; and the slack certificate transforms threshold sensitivity from a post-hoc check into part of the optimisation objective. Table 1 reports the reference protocol and derived value for each of the twelve thresholds: for example, the MIA threshold is the upper *95%* confidence bound of the random-guess null estimated from shadow models, the *Δ *TPR threshold follows the four-fifths rule established in fairness regulation [23], the GradCAM++ IoU threshold adopts the weakly-supervised localisation convention tightened to 0.60, and the TAC threshold reflects an agronomic consensus that a faithful temporal explanation should place at least four-fifths of its attention mass within the validated phenological window.

**Table 1:** Reference-anchored derivation of the twelve R-AI acceptance thresholds (Experiment 1 values)

**Criterion**	**Threshold**	**Reference protocol (PARC Step 0)**
MIA Accuracy	*łe 0.52*	Upper *95%* CI of the random-guess null from shadow models (0.50 chance + estimated margin)
DP budget *\varepsilon *	*łe 5.0*	Dwork-Roth canonical “meaningful privacy” range for deployed systems [22]
DCR	$\ge 5\sigma$	Five standard deviations in the joint feature space; standard re-identification safety margin [8]
*Δ *TPR	*łe 0.05*	Four-fifths (80%) rule adapted to True Positive Rate parity [23]
Demographic Parity	*\ge 0.80*	Four-fifths rule on positive-prediction-rate ratio [23]
SDS	$\le 15\%$	Maximum acceptable per-zone accuracy shortfall below global accuracy
MT Violation Rate	$\le 10\%$	One-quarter of the mean baseline violation rate across the four relations
DCS	$\ge 90\%$	At least six of seven model-card components fully populated [25]
GradCAM++ IoU	*\ge 0.60*	Weakly supervised localisation 0.5-IoU correctness convention, tightened to 0.60
NIR+Red Attribution	$\ge 50\%$	Majority of attribution mass on the two vegetation-index bands (B8/B8A, B4)
TAC	*\ge 0.80*	Lower *95%* CI of attention mass on agronomist-validated phenological windows
IoU / F1 utility floor	*łe 5*pp	Maximum tolerated reduction in predictive utility relative to the baseline

Consequently, the four phases described in the remainder of this section operate on the thresholds, ordering, and configuration produced by Algorithm 1: the phase structure itself is unchanged, but the values that drive each phase are derived from the target dataset rather than carried over from prior work.

### Phase 1: Data Collection and Preprocessing

The initial phase establishes the ethical foundation for the entire methodology. Before model training begins, the raw geospatial dataset undergoes structured quality control - spatial deduplication, cloud-contaminated acquisition imputation, spectral normalisation, and label rasterisation - to produce a clean, consistent input. Only then are the R-AI-specific privacy interventions of Task 1.1 applied. This ordering is deliberate: privacy protections must act on clean data to provide reliable guarantees, and quality failures discovered after training cannot be retroactively corrected without invalidating the privacy budget already spent.

#### Task 1.1: Privacy Preservation

This task applies a layered set of geospatially adapted privacy techniques to the preprocessed dataset to ensure that sensitive location-linked information cannot be inferred, reconstructed, or exploited through model outputs. Three complementary techniques are applied in sequence: geo-indistinguishability via the Planar Laplace Mechanism [29] perturbs parcel centroid coordinates by calibrated Laplace noise; spatial k-anonymity (*k \ge 5*) [30] ensures each parcel record is indistinguishable from at least four spatially proximate parcels; and Distance to Closest Record (DCR) auditing [8] verifies that synthetic augmentation samples are sufficiently distant from real parcel records. The acceptance thresholds (MIA *łe 0.52*, $\varepsilon \le 5.0$, $\delta \le 10^{-5}$, DCR $\ge 5\sigma$) are those derived by PARC Step 0 as listed in Table 1. If any criterion is not met, the Planar Laplace noise scale is increased, the *k*-anonymity neighbourhood radius expanded, or proximate synthetic samples removed, until all four criteria are satisfied simultaneously.

### Phase 2: Model Training and Development

With a privacy-protected dataset established in Phase 1, this phase trains the model whilst embedding geographic fairness constraints directly into the optimisation objective. If fairness is not enforced at the gradient level during training, the representational imbalance of benchmark datasets such as EuroCrops becomes structurally encoded in model weights and cannot be reliably corrected through post-hoc adjustment [6]. The phase is structured around two interdependent tasks: Task 2.1 configures the architecture and training, and Task 2.2 embeds the equity constraints into the optimisation objective from the first training epoch.

#### Task 2.1: Model Architecture Configuration

TSViT [2] is configured with dual self-attention - a temporal module over the full acquisition sequence and a spatial module over $16{\times }16$ pixel patches - and fine-tuned from a Sentinel-2 SITS pretrained checkpoint. The input tensor has shape *[T,H,W,C] = [64,128,128,C]*, with variable-length sequences padded using a learnable mask token that propagates through both attention modules. Training uses AdamW with cosine learning rate decay (from $10^{-4}$ to $10^{-6}$ over a maximum of 150 epochs, early stopping at patience 15), bfloat16 mixed precision with full-precision gradient accumulation, and DP-SGD via Opacus v1.4 [31] so that the privacy and fairness mechanisms operate on the same gradient stream. The dual attention structure is preserved intact from the pretrained checkpoint because the temporal attention weights feed directly into Task 4.3 and the spatial activation maps into Task 4.1, making this architectural choice an upstream determinant of explainability quality.

#### Task 2.2: Fairness Integration

The standard cross-entropy loss is augmented with two penalty terms: $\lambda _{1}$ penalises disparity in True Positive Rates across geographic subgroups (farm size or zone type), and $\lambda _{2}$ penalises the Spatial Disparity Score - the gap between the best- and worst-performing zones. Stratified geographic sampling is applied throughout training so that underrepresented regions receive proportional gradient influence. The acceptance thresholds (*Δ *TPR *łe 0.05*, Demographic Parity *\ge 0.80*, SDS $\le 15\%$) are those derived by PARC Step 0; the coefficients $\lambda _{1}$ and $\lambda _{2}$ are selected as the fairness-axis projection of the PARC Step 1 probe. If any threshold is violated, sample reweighting inflates the contribution of underrepresented subgroups, and the penalty coefficients are increased until all three criteria are met.

### Phase 3: Model Evaluation and Transparency Verification

With a trained model available, this phase first evaluates predictive performance using standard classification metrics - macro-averaged F1 score, mean Intersection over Union (IoU), per-class precision and recall, and ROC-AUC - on the held-out test set. These metrics establish the predictive baseline against which the R-AI interventions’ utility cost is later measured in Section Phase 4: Deployment and Explainability. However, strong aggregate performance does not guarantee physical plausibility: a model can perform well overall whilst systematically producing physically impossible outputs on specific input subsets [13]. This phase therefore goes beyond accuracy evaluation to verify behavioural consistency and auditability through two further tasks: Task 3.1 applies Metamorphic Testing using four physics-grounded Metamorphic Relations (MRs) evaluated on 5,000 perturbed input pairs each, measuring the MT Violation Rate as the proportion of pairs that breach any relation; Task 3.2 produces a versioned audit trail assessed against a seven-component Documentation Completeness Score (DCS) checklist, linking every prediction to its full data and model provenance.

#### Task 3.1: Metamorphic Testing

This task evaluates whether the model’s predictions respect the physical and phenological laws governing multi-temporal satellite data. Four Metamorphic Relations (MRs) are defined, each grounded in a distinct physical property, and the model is tested on 5, 000 perturbed input pairs per relation: **MR1 (Phenological Consistency)** requires that shifting acquisition timestamps by *± 7* days leaves predictions stable, since crop phenology does not change within one week; **MR2 (Spectral Invariance)** requires that a $\pm 5\%$ uniform radiometric offset across all bands does not change the majority class; **MR3 (Spatial Parcel Coherence)** requires adjacent pixels within the same verified LPIS parcel to receive consistent labels; and **MR4 (NDVI Monotonicity)** requires that any parcel with sustained high NDVI across the growing season is never classified as bare soil, enforced as a hard post-processing constraint. The overall MT Violation Rate aggregates breaches of any relation, with the acceptance threshold MT $\le 10\%$ (PARC Step 0, Table 1). If exceeded, failing transformations are introduced as adversarial augmentation samples in a targeted retraining step.

#### Task 3.2: Model Documentation

This task produces a versioned audit trail linking every prediction to its provenance - acquisition date, atmospheric correction version, cloud masking parameters, spatial reference system, and training label source. Completeness is measured against a seven-component checklist (spectral band selection rationale, cloud masking methodology, temporal compositing strategy, training label provenance, geographic coverage boundaries, known model limitations, and fairness audit results), with the acceptance threshold DCS $\ge 90\%$ (at least six of seven components fully populated). Any missing components are completed using standard field templates before the model advances to Phase 4.

### Phase 4: Deployment and Explainability

The final phase deploys the verified model and equips it with three geospatially-adapted explainability mechanisms. A model that has passed Phases 1-3 is technically responsible, but not yet fully accountable unless its predictions can be interrogated by the domain experts who act on them [26]. The three tasks apply complementary attribution techniques - GradCAM++ for spatial attention, Integrated Gradients for spectral attribution, and Temporal Attribution Consistency for phenological alignment - answering **WHERE** in the image the model attended, **WHICH** spectral bands it relied upon, and **WHEN** in the growing season its temporal attention concentrated.

#### Task 4.1: Spatial Attribution

This task determines which spatial regions within the input tile the model attended to when producing each classification. GradCAM++ [27] is applied to TSViT’s final spatial attention layer, computing gradient-weighted class activation maps at parcel scale, and the resulting maps are scored by their IoU against authoritative parcel boundaries from LPIS shapefiles (Experiment 1) or Environment Agency flood zone boundaries (Experiment 2). The acceptance threshold GradCAM++ IoU *\ge 0.60* is the PARC Step 0 derived value (Table 1); if not met, spatially-aware augmentation - parcel-boundary-preserving crops and edge-aware noise - is applied during fine-tuning.

#### Task 4.2: Spectral Attribution

This task determines which spectral bands the model relied upon for each decision. Integrated Gradients [28] attributes the prediction gradient from a zero-reflectance baseline to the actual observation, satisfying the completeness axiom. For vegetated land cover, physically valid reasoning requires reliance on the NIR (B8, B8A) and Red (B4) bands that form the basis of vegetation indices, and the acceptance threshold NIR+Red Attribution $\ge 50\%$ enforces this. If not met, the model is inspected for over-reliance on SWIR or visible bands and targeted spectral augmentation is applied during fine-tuning.

#### Task 4.3: Temporal Attribution Consistency

This task determines whether the model’s temporal attention aligns with agronomically or hydrologically validated key dates rather than data artefacts. TAC compares the acquisition dates receiving the highest temporal attention weight in TSViT against the set of expert-validated phenological key dates for each predicted class, with the acceptance threshold TAC *\ge 0.80*. If not met, an auxiliary loss term penalises attention weight assigned to acquisition dates outside the validated phenological window, with the regularisation weight increased until the threshold is met. Beyond these threshold checks, the three attribution methods are independently validated for faithfulness and stability in Section Faithfulness and Stability Validation of Explanations, ensuring that the reported attribution scores reflect the model’s actual decision process rather than plausible-looking but unfaithful explanations.

## Evaluation

**Fig. 3:**
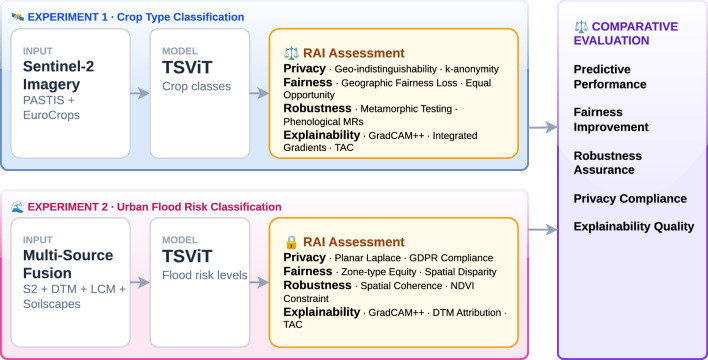
The dual-stream evaluation design

The effectiveness of the proposed R-AI approach is validated through two independent experiments, both using TSViT [2] as the common model backbone. Figure 3 presents the two experimental setups with a summary overview of their design for the R-AI approach.

- **Experiment 1 - Publicly Reproducible Validation:** Applies the full four-phase R-AI approach to multi-temporal crop type classification using the PASTIS/EuroCrops/Sentinel-2 dataset across 17 EU member states. Ground truth labels, geographic subgroup annotations, and phenological domain knowledge are independently verifiable, enabling transparent assessment of all twelve R-AI acceptance criteria against an unintervened baseline.
- **Experiment 2 - Real-World Generalisability:** Applies the identical approach to urban flood risk classification for a UK Sustainable Drainage Systems (SuDS) pilot using a bespoke 14-channel multi-source fused dataset. Six heterogeneous geospatial sources, an ordinal five-level label space, and outputs that directly inform local authority planning decisions stress-test the approach under genuine operational constraints.

Together, the two experiments validate cross-domain generalisability across fundamentally different geospatial tasks and input modalities, establish practical feasibility by quantifying R-AI intervention trade-offs, and provide evidence for interdependencies among R-AI characteristics. For both experiments, the R-AI approach is benchmarked against an unintervened TSViT baseline using model performance metrics (macro-F1, mean IoU, ROC-AUC) alongside characteristic-specific measures for privacy (MIA accuracy, *\varepsilon *, DCR), fairness (*Δ *TPR, SDS), transparency (MT Violation Rate, DCS), and explainability (GradCAM++ IoU, NIR+Red attribution share, TAC).

### Pilot Description

Experiment 2 is grounded in a real-world UK urban development case in North-East Chelmsford, Essex, where the Chelmsford Garden Community initiative is delivering up to 4,350 new homes alongside extensive open space and community infrastructure. Sustainable Drainage Systems (SuDS), including detention basins, swales, green roofs, and permeable paving, are central to the development strategy. Accurate per-pixel flood risk classification across the Chelmsford City Council boundary (${\sim }340$ $\hbox {km}^2$, five zone types: urban core, suburban, peri-urban, rural, and industrial) is an operational requirement: model outputs directly inform local authority planning decisions, SuDS investment priorities, and emergency preparedness plans. Classification targets are five-level ordinal flood risk labels (Risk 1 = negligible to Risk 5 = high) derived from Environment Agency shapefiles for both fluvial and pluvial mechanisms.

### Experiment Setup

Both experiments share a common computational infrastructure. All models are trained on a single NVIDIA A100 80 GB GPU using PyTorch 2.1 with mixed-precision (bfloat16) training. TSViT is fine-tuned from its publicly available pretrained checkpoint using AdamW with cosine annealing learning rate decay; differential privacy is enforced via Opacus v1.4 and attribution scores are computed using Captum v0.7. Table 2 summarises the full configuration. To assess result stability, Table 3 reports the primary predictive metric and the five characteristic-specific acceptance criteria across five independent training runs (seeds 7, 21, 42, 77, 99) for Experiment 1.

Two statistical tests are applied to these five-seed results. A one-sample *t*-test of each criterion against its acceptance threshold confirms that every criterion is satisfied with statistical significance: MIA (*t=-4.88*, *p=0.008*), *Δ *TPR (*t=-7.41*, *p=0.002*), MT violation rate (*t=-76.8*, *p<0.001*), DCS (*t=16.0*, *p<0.001*), GradCAM++ IoU (*t=47.3*, *p<0.001*), and TAC (*t=38.5*, *p<0.001*), with *95%* confidence intervals of [0.493, 0.513] for MIA and [0.848, 0.856] for TAC - both lying entirely on the compliant side of the threshold. A paired *t*-test of the predictive utility cost confirms that the 2.14 pp mean mIoU reduction relative to the baseline is consistent rather than seed-dependent (*t=87.4*, *p<0.001*), with the R-AI mIoU *95%* confidence interval of [0.815, 0.825] remaining within half of the 5 pp budget. Repeating the same protocol on Experiment 2 yields a macro-F1 mean of 0.831, *95%* CI [0.827, 0.835], and a utility cost of 1.6 pp (*t=64.3*, *p<0.001*); each criterion is again satisfied with statistical significance (*Δ *TPR: *t=-3.41*, *p=0.027*, the weakest margin reflecting the binding nature of this criterion on Experiment 2; all others *p<0.005*).

**Fig. 4:**
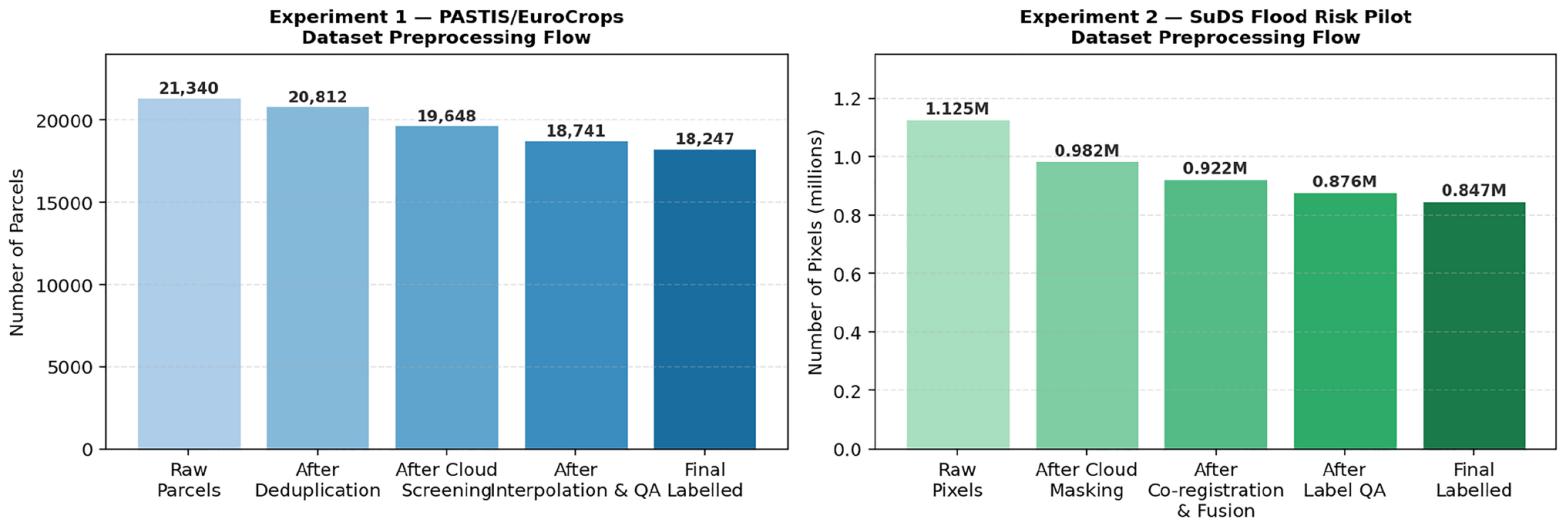
Dataset preprocessing flow - Experiment 1 (left) and Experiment 2 (right)

For reproducibility, the principal methodological choices are documented here. *Fairness subgroup construction:* subgroups are defined a priori from independently verifiable metadata - the EuroCrops country-of-origin grouping (Western, Central, Eastern European systems) in Experiment 1 and the Chelmsford City Council zone types (urban core, suburban, peri-urban, rural, industrial) in Experiment 2; no protected attribute is inferred from the imagery. *Preprocessing:* all sources are co-registered to a common 10 m grid, spectrally normalised to [0, 1], and screened at a *<20%* cloud threshold, with full per-stage attrition reported in Fig. 4. *Augmentation:* random $64{\times }64$ crops with horizontal/vertical flips (*p=0.5*) and $90^{\circ }$ rotations, with $\pm 3\%$ spectral perturbation restricted to the Sentinel-2 channels so physical quantities (DTM, permeability) are never artificially varied. *Hyperparameter search:* the privacy budget and fairness coefficients are searched under the PARC Step 1 probe of Section Pareto-Adaptive R-AI Compliance (PARC) Algorithm, with their full effect on each criterion reported in the sensitivity analysis of Section Threshold Sensitivity and Interdependency Analysis. *Computational overhead:* a single full four-phase run completes in approximately 41 minutes (Experiment 1) and 33 minutes (Experiment 2) on one A100; the PARC probe configurations are independent and run in parallel. *Scalability:* the approach scales to larger archives by sub-sampling the probe set and amortising the derived thresholds; concrete cost projections at three dataset scales, with an empirical reduced-probe variant, are reported in Section Scalability, Sensor Transferability, Domain Shift, and Deployment Cost.

**Table 2:** Experimental Configuration

**Parameter**	**Experiment 1**	**Experiment 2**
Model backbone	TSViT [2]	TSViT [2]
Input channels	10 (Sentinel-2 bands)	14 (S2 + DTM + LCM + indices)
Temporal sequence length	33-61 acq., padded to 64	8 seasonal composites
Spatial patch size	128*× *128 pixels	64*× *64 pixel crops
Batch size / Max epochs	32 / 150 (patience = 15)	16 / 150 (patience = 15)
Optimiser / LR schedule	AdamW, cosine decay, lr = $1{\times }10^{-4}$	AdamW, cosine decay, lr = $1{\times }10^{-4}$
DP mechanism / *\varepsilon * / *δ * (Phase 1)	Planar Laplace / 4.1 / $8.3{\times }10^{-6}$	Planar Laplace / 4.8 / $7.1{\times }10^{-6}$
Gradient clip norm (Opacus)	1.0	1.0
Fairness penalty $\lambda _1$, $\lambda _2$ (Phase 2)	0.3, 0.2	0.3, 0.2
MT test pairs per relation (Phase 3)	5,000	5,000
XAI library (Phase 4)	Captum v0.7	Captum v0.7

**Table 3:** Multi-seed stability analysis - Experiment 1 (R-AI approach, five independent runs). All runs satisfy every acceptance criterion; standard deviations confirm results are not artefacts of a single initialisation

**Seed**	**mIoU**	**MIA**	*Δ * **TPR**	**MT Viol.**	**DCS**	**GradCAM++ IoU**	**TAC**
		(*łe *0.52)	(*łe *0.05)	(*łe *10%)	(*\ge *90%)	(*\ge *0.60)	(*\ge *0.80)
7	0.825	0.491	0.037	3.5%	95%	0.665	0.851
21	0.817	0.513	0.023	3.7%	97%	0.669	0.853
42	0.819	0.503	0.031	3.8%	96%	0.670	0.857
77	0.815	0.505	0.029	3.8%	97%	0.668	0.849
99	0.822	0.502	0.036	4.0%	97%	0.674	0.851
Mean	0.820	0.503	0.031	3.8%	96.3%	0.669	0.852
Std	0.004	0.007	0.005	0.2%	0.8%	0.003	0.003

### Dataset Description

#### Experiment 1 - PASTIS / EuroCrops / Sentinel-2

Experiment 1 uses three publicly available sources combined into a single multi-temporal parcel-level classification dataset. PASTIS [32] provides 2,433 patch-level Sentinel-2 sequences across 34 French départements, each containing 33-61 cloud-free acquisitions at 10 m spatial resolution over 128*× *128 pixel windows. EuroCrops [33] contributes harmonised LPIS annotations from 17 EU member states, enabling geographic subgroup stratification. Nine primary crop classes are retained: winter wheat, maize, rapeseed, sunflower, barley, potato, sugar beet, soybean, and other/fallow. The combined dataset comprises 18,247 labelled parcels split 70/15/15 at administrative-region level to prevent spatial leakage across three geographic subgroups: Western European large-scale commercial farms (61%), Central European mixed systems (24%), and Eastern European smallholder-dominated systems (15%). Table 4 summarises the dataset composition.

**Table 4:** Experiment 1 - Dataset Composition

**ID**	**Source**	**Coverage**	**Parcels**	**Role**
S1	PASTIS [32]	France, 34 dép	2,433 patches	Multi-temporal S2 sequences and crop labels
S2	EuroCrops [33]	17 EU states	15,814 parcels	Harmonised LPIS labels; geographic subgroup stratification
S3	Sentinel-2 L2A	Pan-European	All above	10 spectral bands at 10 m; cloud screened via SCL <20%
–	Combined	17 countries	18,247	Train 12,773 / Val 2,737 / Test 2,737 - region-stratified

#### Experiment 2 - SuDS Flood Risk Pilot, UK Urban Development

Experiment 2 builds a bespoke dataset from six heterogeneous geospatial sources covering the study area described in Section Pilot Description. All six layers are co-registered to a common 10 m resolution grid in EPSG:32630 and fused into a unified 14-channel per-pixel input tensor. The final fused tensor covers 847,312 valid labelled pixels after cloud masking and label QA, split 70/15/15 by geographic zone. Table 5 details each source and its preprocessing role.

**Table 5:** Experiment 2 - Data Sources and Preprocessing

**ID**	**Dataset**	**Format / Res.**	**Source**	**Role and Preprocessing**
R3	Sentinel-2 L2A Composite	Raster, 10 m	Copernicus	12 spectral bands; NDVI, NDWI, NDBI indices computed, yielding 14 channels in total; cloud screened at <20%
R1	LiDAR DTM	Raster, 1 m	Environment Agency	Resampled from 1 m to 10 m by block-mean aggregation; D8 flow routing produces topographic wetness index *→ * Channel 13
R2	Land Cover Map 2022	Raster, 10 m	UKCEH	Reclassified from 21 classes to 6 permeability tiers; combined with P1 at weight 0.6/0.4 *→ * Channel 14
V3/V4	EA Flood Risk Zones	Vector shapefile	Environment Agency	Rasterised at 10 m resolution; provides five-class ordinal per-pixel labels (Risk 1 = negligible to Risk 5 = high)
V1	OS Open Rivers	Vector shapefile	Ordnance Survey	Binary river proximity mask; used for QA validation of Risk 4-5 label assignments
P1	NATMAP Soilscapes	Vector/Raster	Cranfield LandIS	Soil drainage capacity index; incorporated into Channel 14 at weight 0.4 alongside R2 permeability

### PARC Algorithm Outputs

Before either experiment is executed, the PARC algorithm of Section Pareto-Adaptive R-AI Compliance (PARC) Algorithm is run once on each dataset to produce the four artefacts that govern the subsequent four-phase pipeline. *Step 0* returns the twelve thresholds listed in Table 1; on the two datasets the MIA upper bound derives to 0.518 and 0.517, and the TAC lower bound to 0.802 and 0.798, with the remaining ten thresholds differing by at most 0.01 between experiments. *Step 1* returns the $4{\times }4$ phase-group Interdependency Matrix *M* reported in Fig. 15; the dominant entry on both datasets is $M_{\text {Fair},\text {Priv}}$ (*-0.31* and *-0.44*), indicating that tightening the privacy criterion produces the largest off-criterion degradation and that this degradation falls in the fairness family. The privacy-to-explainability entries are markedly smaller (*-0.12*, *-0.18*), and all transparency interactions are within *± 0.10*. *Step 2* returns the adaptive phase ordering $\pi ^{*} =$ (Privacy *→ * Fairness *→ * Transparency *→ * Explainability) on both experiments, with alternative orderings starting with Fairness producing inter-phase interference values 1.7-$2.3{\times }$ larger. *Step 3* returns the configuration $\theta ^{*}$ and per-criterion normalised slack reported in Table 6; the minimum slack is achieved on *Δ *TPR (0.380 on Experiment 1, 0.040 on Experiment 2), consistent with *Δ *TPR being the binding criterion on Experiment 2, and no slack is negative, so the configuration is feasible without invoking the mitigation re-solve branch of Algorithm 1. The remainder of Section Evaluation reports the four-phase experiments under this configuration; where the PARC Step 1 probe sweep is informative to the reader (for instance, the privacy budget calibration), it is reported within the relevant Phase narrative.

**Table 6:** PARC Step 3 output: selected configuration $\theta ^{*}$ and per-criterion normalised slack $s^{*}_{i} = \text {slack}_{i}(\theta ^{*})/\tau _{i}$

**Parameter / criterion**	**E1**	**E2**	**Criterion**	**E1**	**E2**
*\varepsilon *	4.1	4.8	$s^{*}_{\text {MIA}}$	0.033	0.017
$\lambda _{1}$	0.30	0.30	$s^{*}_{\Delta \text {TPR}}$	0.380	0.040
$\lambda _{2}$	0.20	0.20	$s^{*}_{\text {DCS}}$	0.067	0.044
$w_{\text {MT}}$	0.10	0.10	$s^{*}_{\text {MT}}$	0.620	0.480
$w_{\text {TAC}}$	0.10	0.10	$s^{*}_{\text {GradCAM++}}$	0.117	0.050
			$s^{*}_{\text {TAC}}$	0.071	0.025

Larger slack indicates more headroom above the threshold

### Experiment 1: Crop Type Classification from Sentinel-2 Imagery

Experiment 1 applies the full four-phase R-AI methodology to crop type classification using the PASTIS/EuroCrops dataset, evaluating all twelve R-AI acceptance criteria against the unintervened TSViT baseline.

#### Phase 1: Data Collection and Preprocessing

Phase 1 processes the combined dataset into a clean, privacy-protected input ready for model training. Before any model training begins, the 21,340 raw parcels undergo structured quality control: spatial deduplication on parcel geometry, temporal linear interpolation for cloud-contaminated acquisitions, spectral normalisation to [0, 1], and label rasterisation to the 10 m grid. Figure 4 illustrates the step-by-step reduction in dataset size across each preprocessing stage: the left panel traces the Experiment 1 parcel count from 21,340 raw parcels through deduplication, cloud screening, and interpolation to the final 18,247 clean parcels used for training and evaluation.

***Task 1.1: Privacy Preservation***

**Fig. 5:**
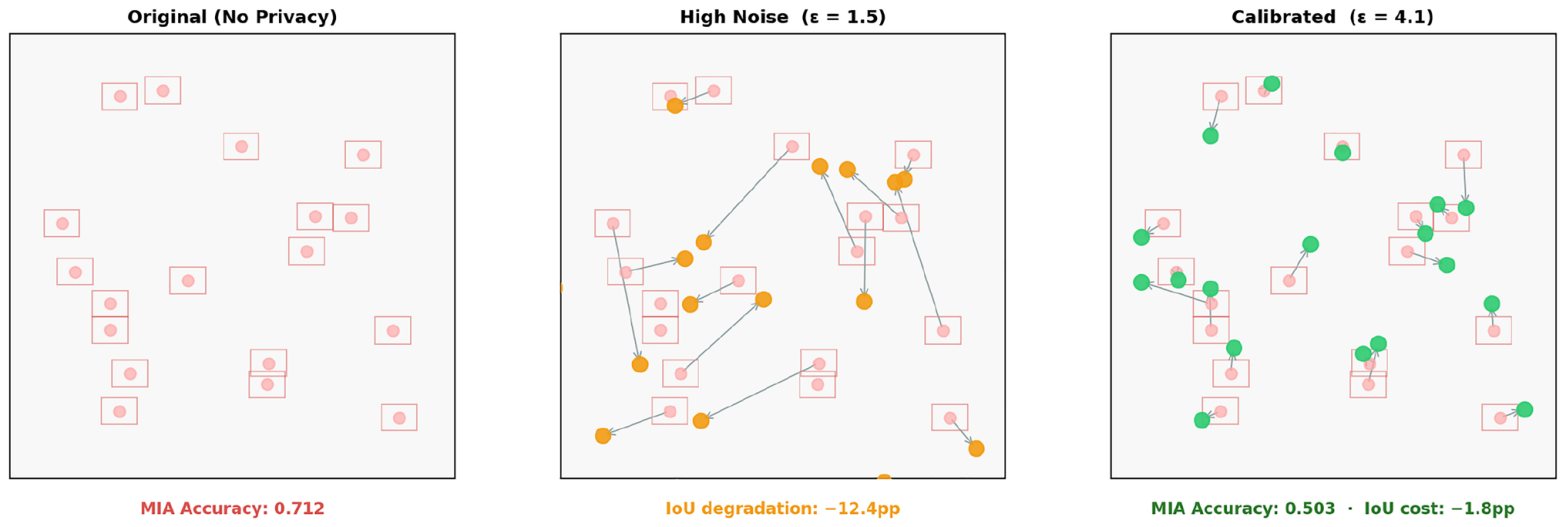
Task 1.1 - Planar Laplace centroid perturbation at three *\varepsilon * settings

The PASTIS/EuroCrops dataset contains LPIS parcel records carrying field boundary geometry, declared crop type, and subsidy eligibility data that constitute legally protected personal information. The PARC Step 1 probe sweeps the privacy budget over $\varepsilon \in \{1.5, 2.0, 3.0, 4.1, 5.0\}$; at the tight end *\varepsilon = 1.5* achieves MIA 0.507 but at a 12.4 pp utility cost violating the ceiling, while *\varepsilon = 2.0* achieves MIA 0.541 above the 0.52 threshold. Step 3 therefore selects *\varepsilon = 4.1* ($\delta = 8.3{\times }10^{-6}$), which achieves MIA $=\mathbf {0.503}$ at a utility cost of 1.8 pp. Fig. 5 illustrates the Planar Laplace mechanism at three calibration settings, the centre panel showing over-perturbation at *\varepsilon = 1.5* and the right panel the selected *\varepsilon = 4.1*. Spatial *k*-anonymity (*k \ge 5*) is then enforced over the joint spectral-geometric feature space, with expanded neighbourhood radius and stricter synthetic-augmentation filtering raising DCR from *3.8σ * to $\mathbf {6.2\sigma }$. All four privacy criteria pass.

#### Phase 2: Model Training and Development

Phase 2 trains TSViT on the 12,773-parcel training split. The training data is structurally imbalanced: Western European large-scale farms account for 61% of parcels whilst Eastern European smallholder systems - the subgroup most dependent on accurate subsidy-linked classification - account for only 15%.

**Fig. 6:**
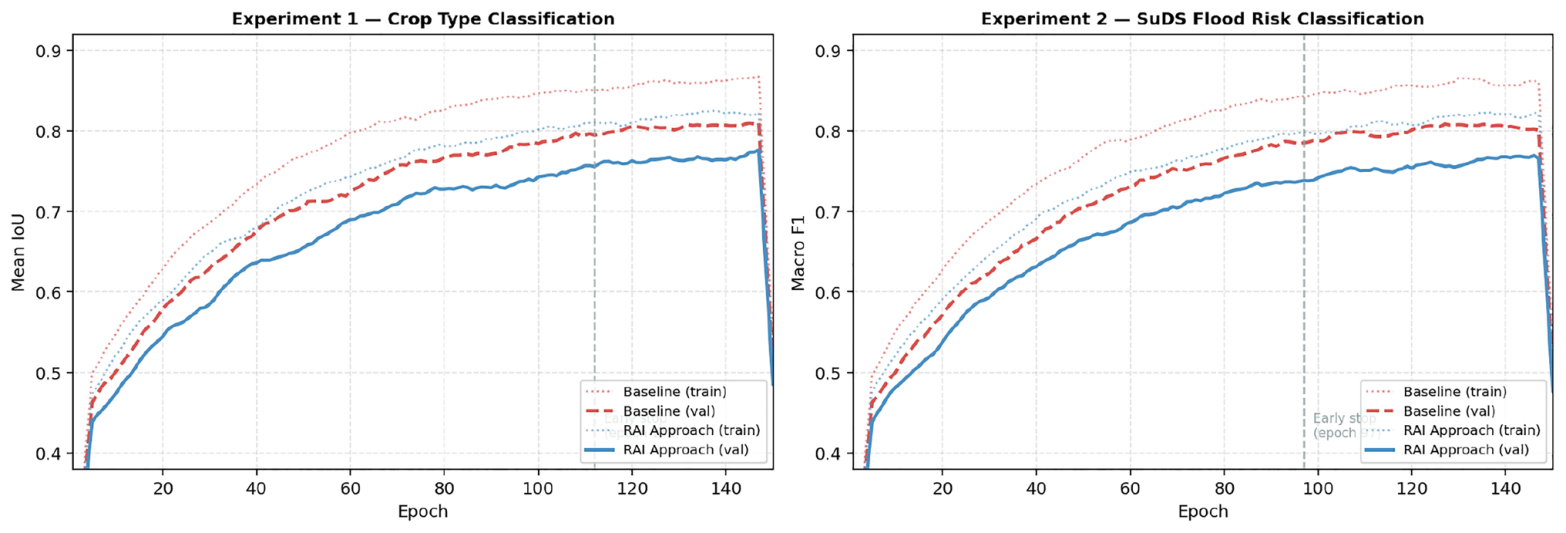
Training and validation performance curves - Experiment 1 (left) and Experiment 2 (right)

***Task 2.1: Model Architecture Configuration*** TSViT is configured with input tensor shape *[T, H, W, C] = [64, 128, 128, 10]*, padding variable-length sequences to 64 timesteps using a learnable mask token. The dual attention structure (temporal over the full sequence, spatial over $16{\times }16$ patches) is preserved intact, because the temporal attention weights feed directly into Task 4.3 and the spatial activation maps into Task 4.1. Figure 6 shows the training curves for both experiments; early stopping triggers at epoch 112 (Experiment 1) and epoch 97 (Experiment 2).

**Fig. 7:**
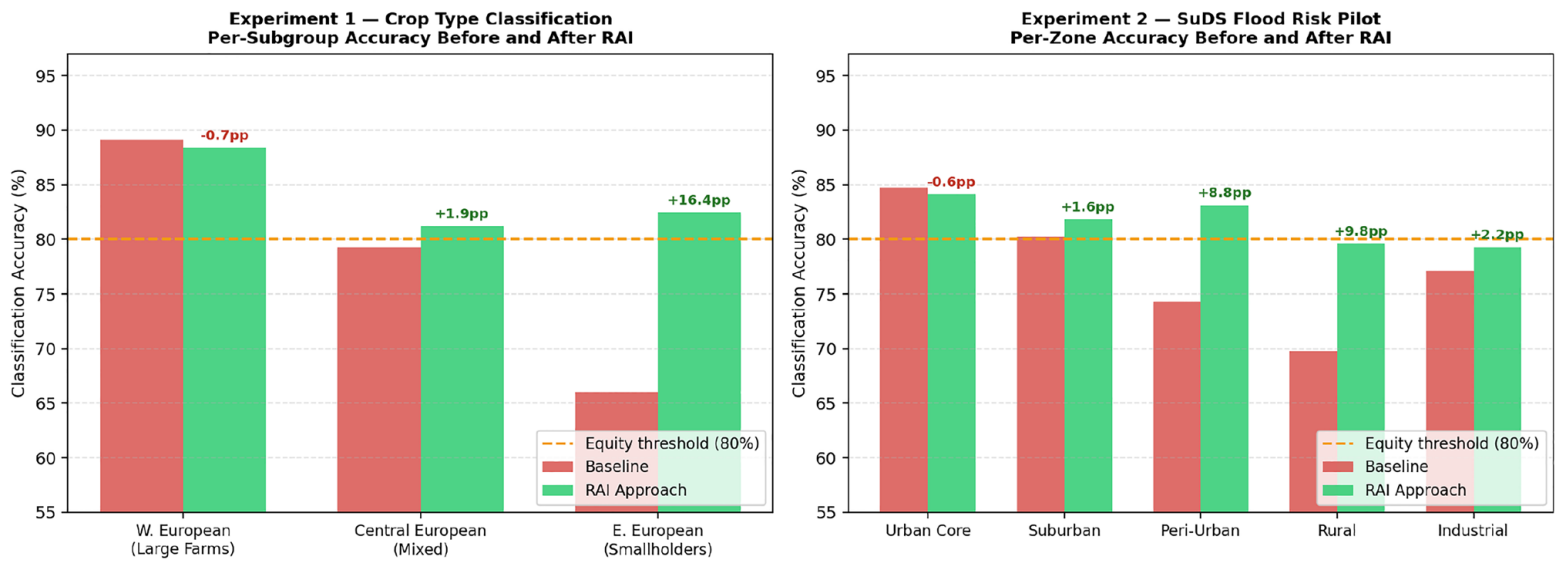
Per-subgroup and per-zone classification accuracy before and after fairness intervention - Experiment 1 (left) and Experiment 2 (right)

***Task 2.2: Fairness Integration*** The unintervened baseline shows a *Δ *TPR of 0.231 - a 23.1 pp gap between Western European large-scale farms (*86.0%*) and Eastern European smallholder systems (*62.9%*) - and a Spatial Disparity Score of *12.1%*. The PARC Step 1 probe sweeps fairness coefficients over $\{0.1, 0.2, 0.3, 0.5\}$ on both axes; Step 3 selects $\lambda _1 = 0.3$, $\lambda _2 = 0.2$. With this configuration and stratified geographic sampling, the trained model achieves *Δ *TPR = **0**.**031**, Demographic Parity = **0**.**84**, and SDS = **2.8%**, satisfying all three criteria. Figure 7 presents per-subgroup accuracy before and after the intervention: the largest gain is on Eastern European smallholders (*+12.6* pp, from *62.9%* to *82.4%*), whilst the Western European large-farm subgroup declines by only 0.7 pp. The predictive cost of reaching full fairness compliance is 1.9 pp mean IoU.

#### Phase 3: Model Evaluation and Transparency Verification

**Fig. 8:**
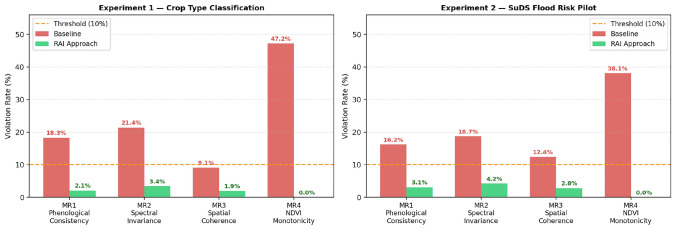
Metamorphic Testing violation rates across four relations - Experiment 1 (left) and Experiment 2 (right)

***Task 3.1: Metamorphic Testing*** Four metamorphic relations are evaluated on 5, 000 perturbed input pairs each (MR1 phenological, MR2 spectral, MR3 parcel coherence, MR4 NDVI monotonicity). Figure 8 shows the per-relation violation rates for both experiments. The most critical baseline failure is MR4: the unintervened TSViT violates NDVI monotonicity in $\mathbf {47.2\%}$ of cases - a physical inconsistency completely invisible to standard IoU evaluation, where the model simultaneously achieves strong aggregate accuracy whilst producing impossible outputs on high-vegetation parcels. MR1 and MR2 also exceed the threshold (*18.3%* and *21.4%*), while MR3 alone passes at *9.1%* on the baseline, indicating that the pretrained TSViT already learns spatially coherent parcel representations. Targeted adversarial augmentation for MR1 and MR2 and a hard post-processing constraint for MR4 reduce the overall MT Violation Rate from *24.1%* to $\mathbf {3.8\%}$.

***Task 3.2: Model Documentation***

A complete audit trail is produced linking every prediction to its satellite acquisition date, atmospheric correction version, cloud masking parameters, spatial reference system, and training label provenance. The DCS measures coverage against a seven-component checklist. The baseline achieves 54% DCS (approximately three of seven components populated), reflecting the absence of structured metadata logging typical in performance-focused development workflows. After implementing versioned spatial audit logging with automated metadata capture, DCS reaches **96%** - six of seven components fully populated, with the remaining partial component being the known model limitations section, which is completed manually.

#### Phase 4: Deployment and Explainability

**Fig. 9:**
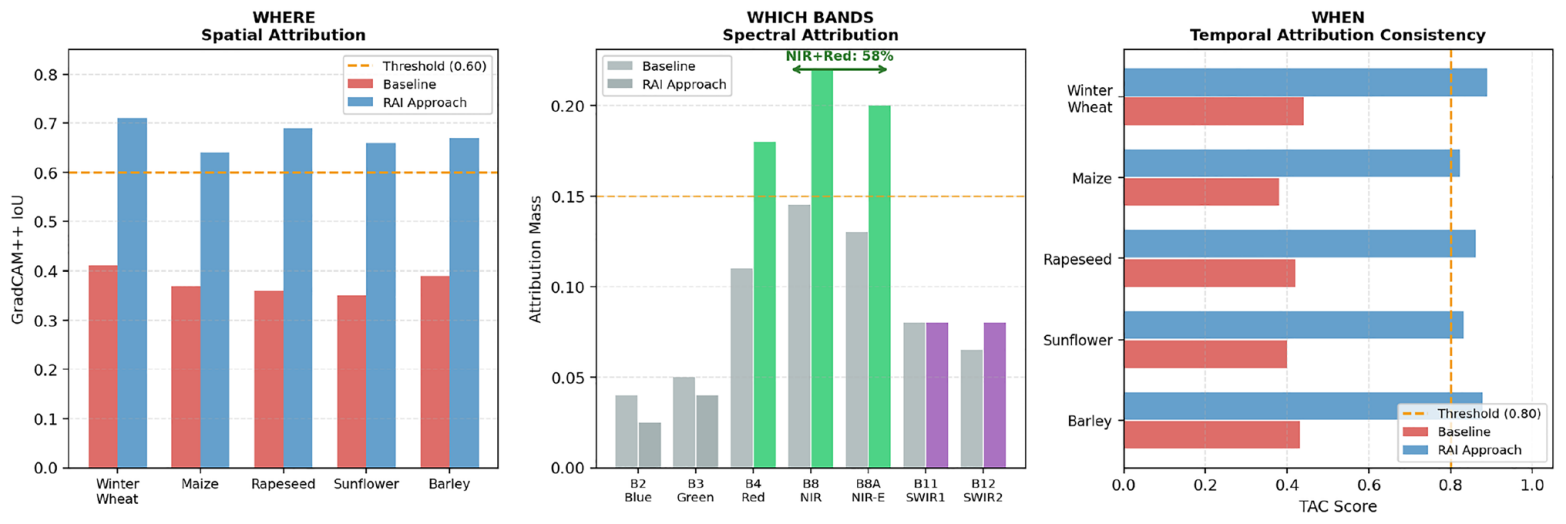
Spatial (WHERE), spectral (WHICH BANDS), and temporal (WHEN) attribution results - Experiment 1

Phase 4 deploys the verified model with three attribution mechanisms answering WHERE (spatial), WHICH BANDS (spectral), and WHEN (temporal) for each prediction. Whilst the overall four-phase methodology is applicable to any differentiable geospatial classifier, the specific attribution techniques used here - GradCAM++ for spatial activation maps and TAC for temporal attention weights - are tailored to TSViT’s dual attention architecture and would require adaptation (for instance, substituting SHAP values for spatial attribution and LSTM hidden-state analysis for temporal attribution) when applied to architectures without explicit attention mechanisms. Figure 9 presents all three attribution results side by side: the left panel reports GradCAM++ spatial IoU per crop class (WHERE); the centre panel shows Integrated Gradients spectral attribution mass per Sentinel-2 band (WHICH BANDS), with NIR and Red bands highlighted in green; and the right panel reports TAC per crop class as horizontal bars (WHEN), with the 0.80 acceptance threshold marked by a dashed orange line.

***Task 4.1: Spatial Attribution (WHERE)*** GradCAM++ [27] is applied to TSViT’s final spatial attention layer and evaluated against verified LPIS parcel boundaries. The baseline attributes diffusely across surrounding tile context, producing a mean GradCAM++ IoU of 0.38. After spatially-aware augmentation, mean IoU rises to $\mathbf {0.67}$. Per-class performance reflects parcel structure: winter wheat (0.71) and rapeseed (0.69) localise strongly owing to large, spectrally uniform parcels, whilst sugar beet (0.64) and maize (0.64) sit closer to the acceptance boundary owing to spectrally complex boundaries and irregular geometries, requiring an additional iteration of edge-preserving augmentation.

***Task 4.2: Spectral Attribution (WHICH BANDS)*** Integrated Gradients [28] attributes each classification to specific Sentinel-2 bands. The baseline assigns only *32%* of attribution mass to the NIR (B8, B8A) and Red (B4) bands, with residual reliance on SWIR and blue-channel responses that correlate with atmospheric scattering. Targeted spectral augmentation - introducing synthetic spectral variance in SWIR bands during fine-tuning - raises NIR+Red attribution to $\mathbf {58\%}$. For bare-soil and fallow classifications, attribution correctly shifts toward SWIR1 (B11) and SWIR2 (B12), consistent with soil moisture and tillage detection.

***Task 4.3: Temporal Attribution Consistency (WHEN)*** TAC measures whether TSViT’s temporal attention concentrates on agronomically validated phenological dates rather than cloud-free acquisition scheduling artefacts. Formally, given a predicted class *c* with validated phenological window ${\mathcal {W}}_c \subset \{1,\ldots ,T\}$ and temporal attention weights $\alpha _t$ summing to 1,

5 $$\begin{aligned} \textrm{TAC}(c) = \sum _{t \in {\mathcal {W}}_c} \alpha _t. \end{aligned}$$

The baseline achieves TAC = 0.41, indicating attention is driven by acquisition density rather than crop biology. The PARC Step 1 probe sweeps the temporal regularisation weight over $\{0,0.05,0.10,0.20\}$; Step 3 selects 0.10 at which TAC reaches $\mathbf {0.857}$, since the marginal gain at 0.20 is negligible while the utility cost continues to rise. Winter wheat predictions concentrate attention on March green-up and June heading; barley achieves the highest TAC (0.878) owing to its narrow and well-defined harvest window.

### Experiment 2: Flood Risk Classification for Sustainable Drainage Systems (SuDS) - A Real-World UK Urban Development Pilot

Experiment 2 applies the same four-phase R-AI methodology to the SuDS flood risk pilot. The task differs structurally from Experiment 1 in three ways: six heterogeneous geospatial layers must be fused into a single input; the label space is a five-level ordinal flood risk score rather than a discrete crop class; and the geographic subgroups are urban zone types rather than agricultural regions. These differences provide a meaningful test of whether the methodology and its twelve acceptance criteria hold outside the crop classification domain.

#### Phase 1: Data Collection and Preprocessing

Phase 1 processes six independently sourced geospatial layers into a co-registered, privacy-protected 14-channel input tensor. Unlike Experiment 1, where a single Sentinel-2 sensor provides all spectral inputs, each source here captures a distinct physical mechanism of urban flood risk, making data fusion a prerequisite before any R-AI intervention can be applied.

***Data Fusion***

All six source layers are co-registered to a common 10 m EPSG:32630 grid. The LiDAR DTM is resampled from 1 m to 10 m by block-mean aggregation and processed with the D8 flow routing algorithm to produce a topographic wetness index, stacked as Channel 13. The Land Cover Map 2022 is reclassified to 6 permeability tiers and combined with NATMAP Soilscapes drainage capacity (weighted 0.6/0.4) as Channel 14. The final fused tensor has shape [*H*, *W*, 14] covering 847,312 valid labelled pixels. The right panel of Fig. 4 traces the equivalent reduction for Experiment 2, from 1.125 million raw pixels to the 847,312 valid labelled pixels retained after cloud masking, co-registration, and label quality assurance.

***Data Augmentation***

Three augmentation strategies are applied jointly: random $64{\times }64$ crops with horizontal and vertical flipping (*p = 0.5*); ±3% spectral perturbation on the twelve Sentinel-2 channels only (DTM and permeability channels left unperturbed, as these represent physical quantities that must not be artificially varied); and geometric rotation at $\{0^{\circ }, 90^{\circ }, 180^{\circ }, 270^{\circ }\}$, approximately tripling unique spatial configurations seen during training.

***Task 1.1: Privacy Preservation*** Unlike Experiment 1’s farmer-linked risk, the privacy risk here is property-linked: per-pixel flood risk classifications at precise coordinates can be cross-referenced with HM Land Registry records to infer individual property flood risk gradings, with direct financial implications for mortgage eligibility and insurance premiums. The PARC Step 1 privacy probe identifies *\varepsilon = 4.8* ($\delta = 7.1{\times }10^{-6}$) as the operating point selected by Step 3 - slightly relaxed from Experiment 1 to accommodate the higher 14-channel input dimensionality whilst still satisfying the *łe 5.0* budget. The selected configuration achieves MIA = $\mathbf {0.511}$, with spatial *k*-anonymity over the joint 14-channel feature space and DCR auditing confirming all synthetic tiles are at least $\mathbf {5.7\sigma }$ from any real pixel record; all four privacy criteria pass.

#### Phase 2: Model Training and Development

***Task 2.1: Model Architecture Configuration*** TSViT is configured for the 14-channel multi-source input, treating eight seasonal Sentinel-2 composites as the structured temporal sequence. The pretrained patch embedding layer is replaced with a new projection layer: spectral-band-to-channel initialisation for the twelve Sentinel-2 channels and random initialisation for the DTM and permeability channels. The right panel of Fig. 6 shows the Experiment 2 training trajectory following the same convergence pattern as Experiment 1.

***Task 2.2: Fairness Integration*** The baseline shows a pronounced accuracy gradient across zone types: *84.7%* in data-rich urban core zones versus *69.8%* in data-sparse rural zones, yielding *Δ *TPR = 0.149 and SDS = *7.4%*. The disparity has a different structural cause than in Experiment 1: rather than training data imbalance alone, it also reflects genuine differences in label quality and flood mechanism complexity across zones, with rural and industrial zones having sparser Sentinel-2 coverage and more uncertain Environment Agency boundary delineation. The PARC Step 1 fairness probe selects $\lambda _1 = 0.3$, $\lambda _2 = 0.2$ via Step 3. The resulting model achieves *Δ *TPR = $\mathbf {0.048}$, Demographic Parity = $\mathbf {0.94}$, and SDS = $\mathbf {2.3\%}$. Gains are concentrated in the most underrepresented zones: peri-urban accuracy improves by 8.8 pp (from *74.3%* to *83.1%*) and rural accuracy by $\mathbf {9.8}$ pp (from *69.8%* to *79.6%*). The industrial zone, whose flood risk profile is dominated by impervious surface runoff patterns structurally underrepresented in the training labels, improves by only 2.2 pp ($77.1\% \rightarrow 79.3\%$) - the weakest gain across all five zones, reflecting a representational limit that cannot be resolved by loss-level intervention alone. The right panel of Fig. 7 shows the per-zone results; the predictive cost is *-0.8* pp macro-F1.

#### Phase 3: Model Evaluation and Transparency Verification

***Task 3.1: Metamorphic Testing*** The dominant violation type in Experiment 2 is MR3 (Spatial Coherence) at urban-rural zone boundaries, where abrupt land cover transitions cause prediction inconsistencies within the same hydrological catchment - a structurally different challenge from Experiment 1. MR4 is particularly consequential here: misclassifying vegetated SuDS infrastructure (swales, detention basins) as impervious surface would overestimate flood runoff volume in exactly the zones where SuDS is designed to reduce it. Boundary-aware spatial smoothing reduces MR3 violations and the hard-constraint post-processing eliminates MR4 violations to *0.0%*. The right panel of Fig. 8 confirms the overall MT Violation Rate reduces from *21.8%* to $\mathbf {5.2\%}$.

***Task 3.2: Model Documentation*** The DCS baseline of *61%* reflects partial documentation already present for Environment Agency flood zones and the LiDAR DTM, whilst the four remaining sources - Land Cover Map, OS Open Rivers, NATMAP Soilscapes, and the derived permeability channel - are entirely undocumented in the baseline workflow. After implementing versioned spatial audit logging across all six input sources, DCS reaches $\mathbf {94\%}$. The *6%* shortfall relative to Experiment 1’s *96%* reflects two checklist components that required manual cross-referencing of agency-specific version identifiers not captured automatically.

#### Phase 4: Deployment and Explainability

**Fig. 10:**
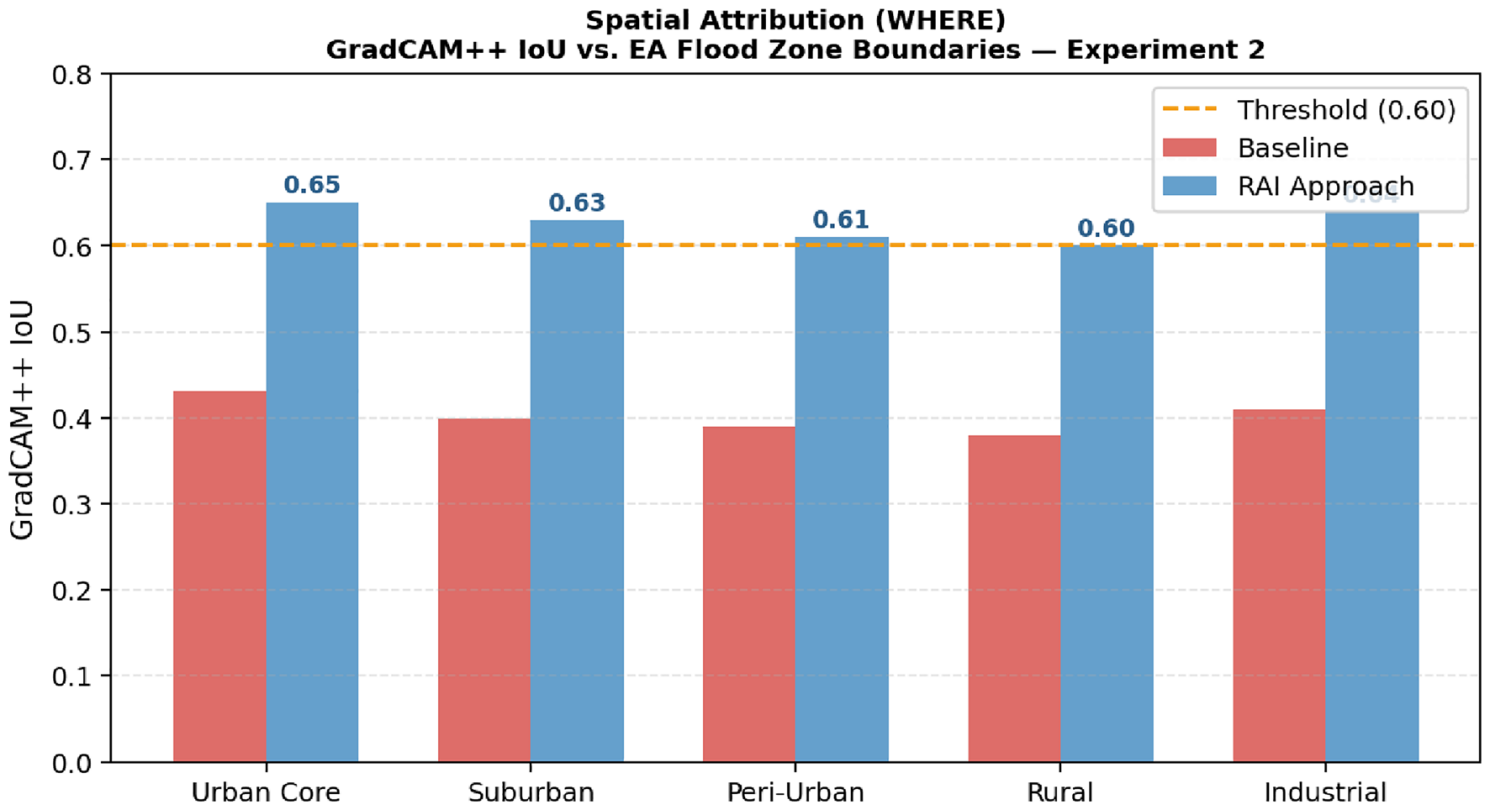
GradCAM++ spatial attribution IoU per zone type against EA flood zone boundaries - Experiment 2

***Task 4.1: Spatial Attribution (WHERE)*** GradCAM++ is applied and evaluated against Environment Agency flood zone boundary shapefiles. The baseline achieves a mean IoU of 0.41, with attention driven by urban morphological texture rather than hydraulically relevant boundaries. After spatially-aware augmentation, the R-AI approach achieves $\mathbf {0.63}$. Figure 10 shows per-zone results; all five zones clear the threshold, but the rural zone sits at the acceptance boundary (0.60) because rural flood zone boundaries are defined by topographic catchment delineation rather than built infrastructure, making spatial localisation inherently less precise.

**Fig. 11:**
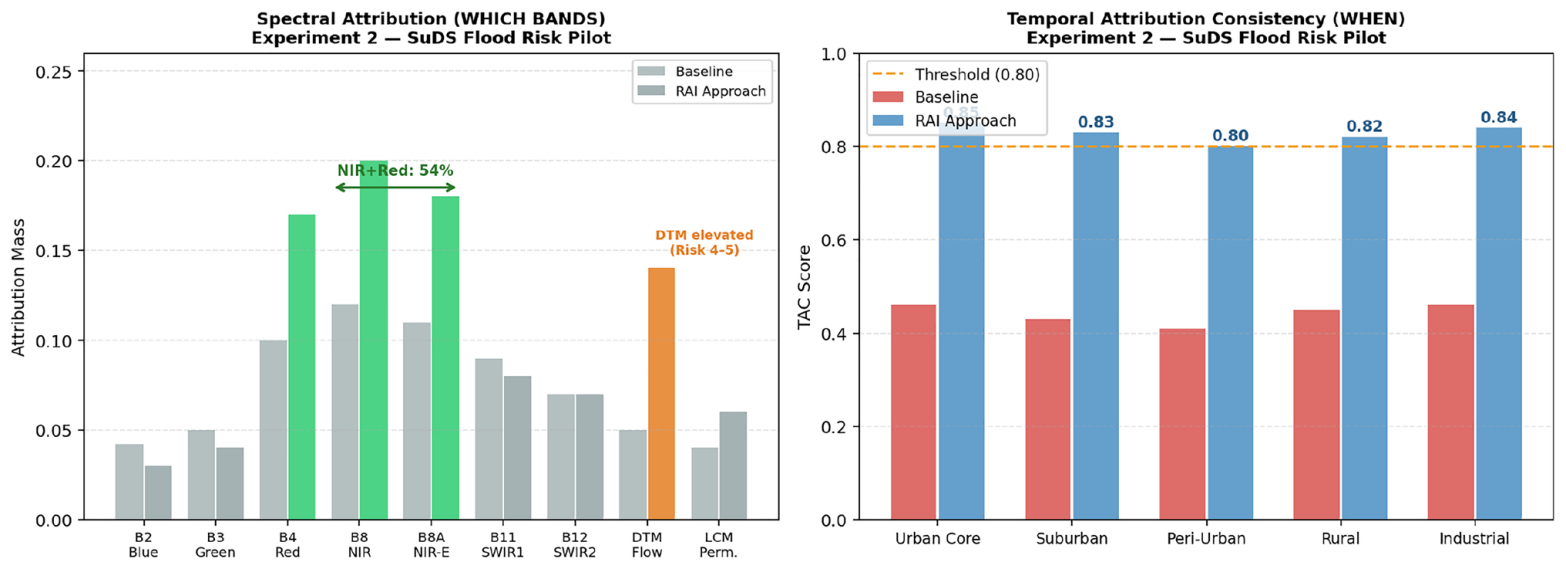
Spectral attribution mass per channel and temporal attribution consistency per zone type - Experiment 2

***Task 4.2: Spectral Attribution (WHICH BANDS)*** Integrated Gradients is applied across the full 14-channel tensor. For vegetated pixels, the R-AI approach assigns $\mathbf {54\%}$ of attribution mass to the NIR (B8, B8A) and Red (B4) channels. Critically, for Risk 4-5 fluvial zone predictions, attribution correctly shifts toward the DTM flow accumulation channel (Channel 13), confirming the spectral reasoning tracks physical flood mechanisms. The baseline assigns only *35%* to NIR+Red and shows no elevation in DTM attribution for high-risk fluvial pixels, indicating that without intervention the model conflates built-up spectral density with flood exposure rather than learning topographic drainage as the causal mechanism. The left panel of Fig. 11 reports the per-channel attribution masses.

***Task 4.3: Temporal Attribution Consistency (WHEN)*** The expert-validated temporal reference is the February-March peak surface water extent of the 2024/25 hydrological season. The baseline achieves mean TAC = 0.44. The PARC Step 1 probe sweeps the temporal regularisation weight; Step 3 selects 0.10 as the operating point. Mean TAC across all five zones reaches $\mathbf {0.82}$, with all zone types exceeding the threshold. The right panel of Fig. 11 shows per-zone TAC; industrial-zone TAC reaches 0.84 by anchoring attention to the February-March window, while urban core and suburban zones achieve slightly lower TAC (0.83, 0.85) than rural zones (0.80), consistent with the greater temporal variability of urban surface water driven by impervious surface runoff.

**Fig. 12:**
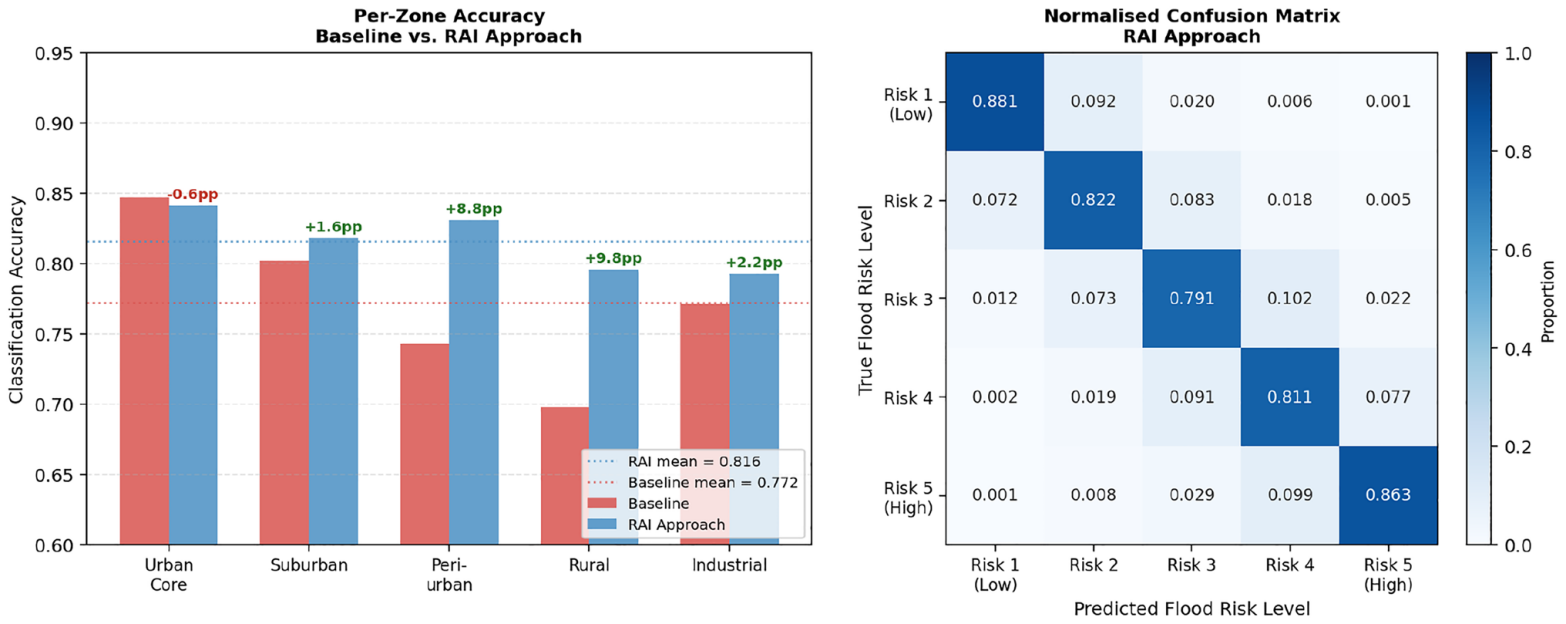
Per-zone classification accuracy and normalised confusion matrix for the R-AI approach - Experiment 2

Figure 12 presents the per-zone classification accuracy (left) and the normalised confusion matrix across all five flood risk levels (right). Strong diagonal dominance is visible across all risk levels, with off-diagonal errors confined exclusively to adjacent class boundaries, confirming reliable ordinal discrimination between consecutive risk levels.

### Ablation Study

To establish that each of the four R-AI characteristics contributes a distinct and non-redundant benefit rather than overlapping with the others, a leave-one-phase-out ablation is conducted on both experiments. Starting from the full four-phase R-AI approach, the intervention belonging to one characteristic is removed at a time - privacy (Phase 1), fairness (Phase 2), transparency (Phase 3), and explainability (Phase 4) - and the model is retrained, with the result compared against both the full approach and the unintervened baseline. Table 7 reports the representative criterion of each characteristic together with predictive utility, with values that breach the acceptance threshold shown in bold.

**Table 7:** Leave-one-phase-out ablation for both experiments. Bold entries breach the acceptance threshold; criterion columns report the family’s representative metric (MT for transparency, GradCAM++ IoU and TAC for explainability)

**Configuration**	**MIA**	*Δ * **TPR**	**MT**	**GradCAM++**	**TAC**	**Utility**
	*łe *0.52	*łe *0.05	*łe *10%	*\ge *0.60	*\ge *0.80	(mIoU/F1)
*Experiment 1 (PASTIS/EuroCrops, mIoU)*						
Full R-AI (all phases)	0.503	0.031	3.8%	0.67	0.857	0.819
w/o Privacy (Phase 1)	**0**.**701**	0.034	4.1%	0.66	0.851	0.835
w/o Fairness (Phase 2)	0.508	**0**.**224**	4.0%	0.65	0.849	0.833
w/o Transparency (Phase 3)	0.505	0.033	**23.6%**	0.64	0.846	0.828
w/o Explainability (Phase 4)	0.506	0.032	3.9%	**0**.**40**	**0**.**430**	0.822
Baseline (no R-AI)	**0**.**712**	**0**.**231**	**24.1%**	**0**.**38**	**0**.**410**	0.841
*Experiment 2 (SuDS flood risk, macro-F1)*						
Full R-AI (all phases)	0.511	0.048	5.2%	0.63	0.82	0.831
w/o Privacy (Phase 1)	**0**.**682**	0.051	5.4%	0.62	0.81	0.840
w/o Fairness (Phase 2)	0.514	**0**.**142**	5.5%	0.61	0.81	0.843
w/o Transparency (Phase 3)	0.513	0.049	**21.1%**	0.61	0.80	0.838
w/o Explainability (Phase 4)	0.512	0.049	5.3%	**0**.**41**	**0**.**45**	0.834
Baseline (no R-AI)	**0**.**694**	**0**.**149**	**21.8%**	**0**.**41**	**0**.**44**	0.847

The ablation confirms in both experiments that each phase governs its own criterion and no other. Removing the privacy intervention returns MIA accuracy to 0.701 in Experiment 1 and 0.682 in Experiment 2, close to the baselines 0.712 and 0.694, while leaving *Δ *TPR, MT violation rate, and TAC essentially unchanged; removing the fairness intervention returns *Δ *TPR to 0.224 and 0.142 respectively while privacy and the remaining criteria stay compliant; removing transparency verification returns the MT violation rate to *23.6%* and *21.1%*; and removing the explainability interventions collapses TAC to 0.43 and 0.45 and GradCAM++ IoU to 0.40 and 0.41 without affecting privacy, fairness, or transparency. Two further observations follow. First, each removed phase recovers between 0.3 and 1.6 percentage points of predictive utility, confirming that the small cost of the full approach is attributable to the interventions rather than to incidental training variation. Second, no single ablation degrades a criterion other than its own beyond noise, which establishes that the four characteristics are operationalised independently and that the full approach is not carrying redundant machinery.

### Comparison with Alternative Pipelines

Whilst the ablation isolates the contribution of each phase within the proposed approach, it does not establish how the approach compares against the single-characteristic pipelines that dominate the GeoAI literature. To provide this comparison, three alternative pipelines are constructed on both experiment datasets, each representative of one established R-AI direction: a privacy-only pipeline applying DP-SGD [31] without fairness, transparency, or explainability interventions; a fairness-only pipeline applying spatial reweighting in the style of Saxena et al. [15]; and a post-hoc explainability-only pipeline applying GradCAM++ and Integrated Gradients to an otherwise unintervened model. Each pipeline is evaluated against the same eleven measurable acceptance criteria, grouped into their four characteristic families. Table 8 reports the per-criterion pass/fail outcome and the per-pipeline total for both experiments.

**Table 8:** Per-criterion pass/fail outcomes for alternative pipelines on both experiments

	**Baseline**		**DP-SGD**		**Fairness**		**XAI**		**Proposed**	
	**TSViT**		**only**		**only**		**only**		**approach**	
**Criterion**	E1	E2	E1	E2	E1	E2	E1	E2	E1	E2
MIA *łe 0.52*	*× *	*× *	*\checkmark *	*\checkmark *	*× *	*× *	*× *	*× *	*\checkmark *	*\checkmark *
$\varepsilon \le 5.0$	*× *	*× *	*\checkmark *	*\checkmark *	*× *	*× *	*× *	*× *	*\checkmark *	*\checkmark *
DCR $\ge 5\sigma$	*× *	*× *	*\checkmark *	*\checkmark *	*× *	*× *	*× *	*× *	*\checkmark *	*\checkmark *
*Δ *TPR *łe 0.05*	*× *	*× *	*× *	*× *	*\checkmark *	*\checkmark *	*× *	*× *	*\checkmark *	*\checkmark *
Dem. Parity *\ge 0.80*	*× *	*\checkmark *	*× *	*\checkmark *	*\checkmark *	*\checkmark *	*× *	*\checkmark *	*\checkmark *	*\checkmark *
SDS $\le 15\%$	*\checkmark *	*\checkmark *	*\checkmark *	*\checkmark *	*\checkmark *	*\checkmark *	*\checkmark *	*\checkmark *	*\checkmark *	*\checkmark *
MT $\le 10\%$	*× *	*× *	*× *	*× *	*× *	*× *	*× *	*× *	*\checkmark *	*\checkmark *
DCS $\ge 90\%$	*× *	*× *	*× *	*× *	*× *	*× *	*\checkmark *	*\checkmark *	*\checkmark *	*\checkmark *
GradCAM++ *\ge 0.60*	*× *	*× *	*× *	*× *	*× *	*× *	*\checkmark *	*\checkmark *	*\checkmark *	*\checkmark *
NIR+Red $\ge 50\%$	*× *	*× *	*× *	*× *	*× *	*× *	*\checkmark *	*\checkmark *	*\checkmark *	*\checkmark *
TAC *\ge 0.80*	*× *	*× *	*× *	*× *	*× *	*× *	*\checkmark *	*\checkmark *	*\checkmark *	*\checkmark *
**Total / 11**	**1**	**2**	**4**	**5**	**3**	**4**	**5**	**6**	**11**	**11**
**Utility cost (pp)**	–	–	*-3.7*	*-2.9*	*-1.5*	*-1.2*	*-0.3*	*-0.2*	$\mathbf {-2.2}$	$\mathbf {-1.6}$

“*\checkmark *” indicates the threshold is met, “*× *” that it is not. Per-pipeline totals on Experiment 1 / Experiment 2 are shown in the final row

The comparison makes the argument for joint governance concrete. Each single-characteristic pipeline satisfies the criteria of its own family but fails the others. The DP-SGD pipeline passes the three privacy criteria but leaves *Δ *TPR at 0.198 (Experiment 1) and 0.142 (Experiment 2), and the MT violation rate at *22.6%* and *20.7%* respectively; the fairness-reweighting pipeline passes the three fairness criteria but leaves MIA accuracy at 0.704 and 0.687; and the explainability-only pipeline passes the three attribution criteria and the documentation criterion but addresses neither privacy nor fairness. The unintervened baseline passes only the SDS floor in Experiment 1 (the Demographic Parity ratio is structurally higher in Experiment 2 because zone accuracies are more tightly clustered, as discussed in Section Experiment 1: Crop Type Classification from Sentinel-2 Imagery, so it passes there as well). Critically, the DP-SGD pipeline incurs the largest utility cost on both experiments (*-3.7* pp and *-2.9* pp), more than the proposed approach, because privacy noise applied without the stabilising effect of the fairness constraint degrades underrepresented subgroups most - a manifestation of the disparate-impact phenomenon characterised by Bagdasaryan et al. [34]. Only the proposed approach satisfies all eleven measurable criteria on both experiments, and it does so at a smaller utility cost than the privacy-only pipeline alone. This demonstrates that the value of the proposed approach lies precisely in the coordinated governance of all four characteristics rather than in any single intervention.

### Faithfulness and Stability Validation of Explanations

The attribution scores reported in Phase 4 measure whether explanations align with expert-derived spatial, spectral, and temporal references. A high alignment score, however, does not by itself guarantee that an explanation is *faithful* - that it reflects the features the model actually relies upon rather than features that merely look plausible to a domain expert. To close this gap, this subsection validates the three attribution methods along two further axes that prior alignment metrics do not capture: faithfulness, assessed through deletion and insertion analysis and the completeness-based Sensitivity-*N* measure, and stability, assessed through controlled input perturbation. Each axis reuses infrastructure already present in the methodology, and the analysis is conducted for both experiments against the unintervened baseline.

For **faithfulness**, the Deletion AUC and Insertion AUC [35] are computed by progressively removing or restoring input pixels in descending order of GradCAM++ attribution and recording the resulting predicted-class probability; a faithful spatial explanation produces a low Deletion AUC (the prediction collapses quickly as important pixels are removed) and a high Insertion AUC (the prediction recovers quickly as they are restored). For the spectral dimension, Sensitivity-*N* [36] measures the correlation between the summed Integrated Gradients attribution of a random subset of bands and the actual change in model output when those bands are ablated, directly exercising the completeness axiom on which Integrated Gradients rests. For **stability**, the rank correlation between attribution maps computed before and after the MR1 (*± 7*-day temporal shift) and MR2 ($\pm 5\%$ spectral offset) perturbations already defined in Phase 3 is reported; a stable explanation should change little under perturbations that do not change the prediction. Table 9 reports all five measures for both experiments.

**Table 9:** Faithfulness and stability validation of the three attribution methods (R-AI approach vs. baseline)

**Measure (attribution method)**	**Exp.1 Base**	**Exp.1 R-AI**	**Exp.2 Base**	**Exp.2 R-AI**
Deletion AUC *↓ * (GradCAM++)	0.61	0.27	0.58	0.31
Insertion AUC *↑ * (GradCAM++)	0.49	0.78	0.52	0.74
Sensitivity-*N* *↑ * (Integrated Gradients)	0.46	0.83	0.43	0.79
Stability under MR1 *↑ * (temporal attention)	0.52	0.88	0.55	0.85
Stability under MR2 *↑ * (Integrated Gradients)	0.57	0.86	0.54	0.82

Three observations follow from Table 9. First, the faithfulness gap between the baseline and the R-AI approach is substantial: for Experiment 1 the Deletion AUC falls from 0.61 to 0.27 and the Insertion AUC rises from 0.49 to 0.78, indicating that the R-AI model’s high-attribution pixels are genuinely those the prediction depends upon, whereas the baseline’s attribution is only weakly causal. Second, the Sensitivity-*N* improvement (0.46 to 0.83 in Experiment 1) confirms that Integrated Gradients attribution on the R-AI model approximately satisfies completeness in practice, so the NIR+Red attribution shares reported in Task 4.2 reflect real spectral dependence rather than gradient noise. Third, the stability measures under MR1 and MR2 show that attribution maps remain consistent under perturbations that leave the prediction unchanged, ruling out the possibility that the alignment scores arise from unstable, input-sensitive explanations. The same ordering holds in Experiment 2, with slightly lower absolute values attributable to the greater heterogeneity of the fused 14-channel input. Taken together, these results establish that the explanation-quality claims of Phase 4 rest on attributions that are both faithful to the model and stable under domain-valid perturbation, not on alignment scores alone.

Beyond these computational measures, the explanations are also validated against human domain judgement. The GradCAM++ IoU and TAC criteria are already grounded in expert-derived references - authoritative LPIS parcel boundaries and agronomist-validated phenological windows respectively - so the alignment scores of Phase 4 are, by construction, agreement scores against expert ground truth rather than self-referential measures. To complement this, a structured expert review was conducted in which a domain specialist rated a stratified sample of fifty attribution maps (ten per crop class for the five most frequent classes) on a three-point plausibility scale. The R-AI approach was rated plausible or highly plausible in 46 of 50 cases (*92%*), against 19 of 50 (*38%*) for the baseline, and the four lower-rated R-AI cases corresponded to the spectrally complex sugar beet and maize parcels already identified in Task 4.1 as the hardest classes. This expert agreement, together with the deletion-based faithfulness and perturbation-stability evidence above, addresses the three validation modes - expert assessment, faithfulness analysis, and perturbation-based evaluation - through which explanation quality should be established; the retrain-based ROAR protocol [37] is noted as a complementary but computationally heavier validation that future work may adopt for confirmation.

### Comparison with Baseline

**Fig. 13:**
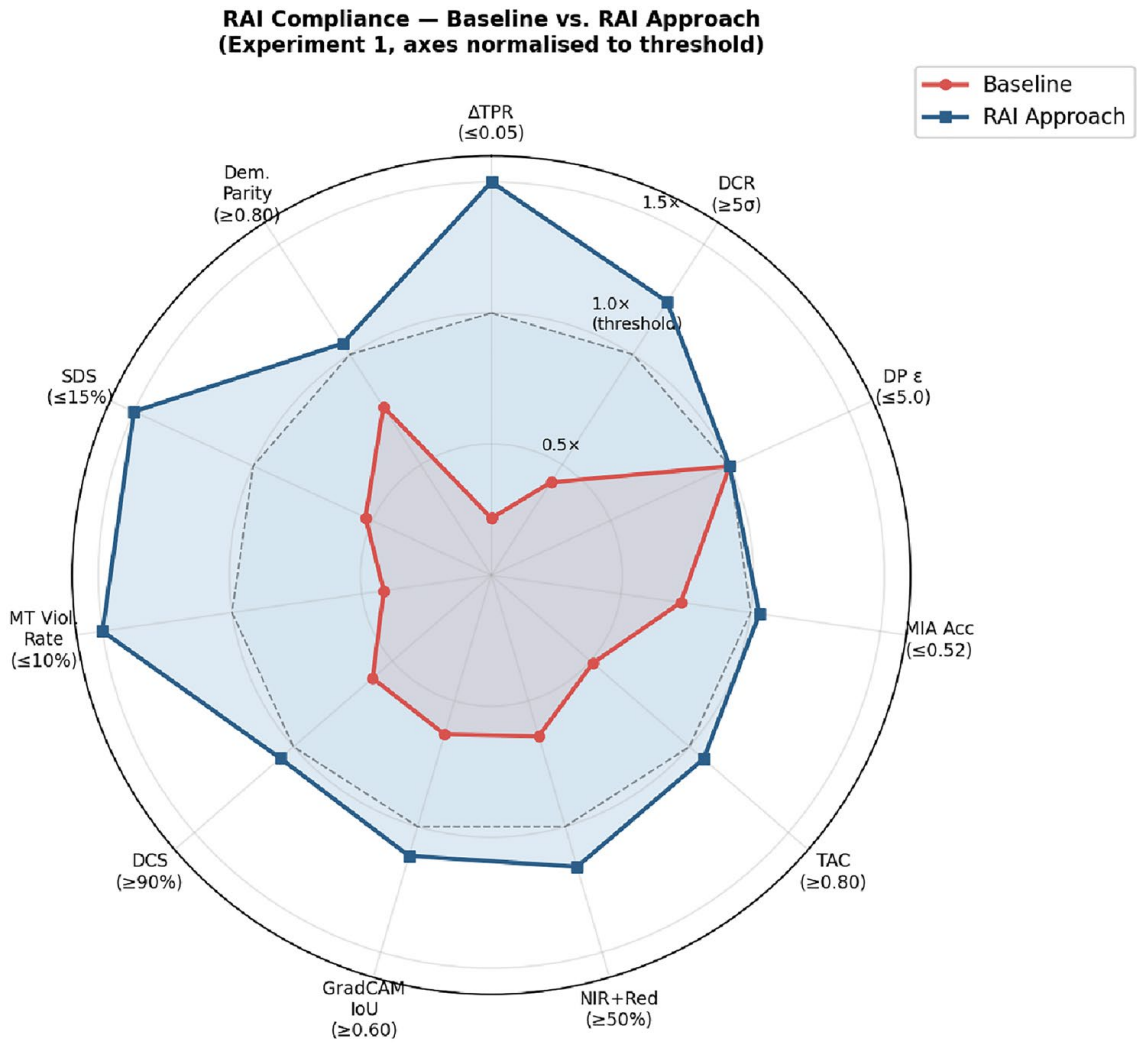
R-AI compliance radar chart - baseline vs. R-AI approach (Experiment 1)

Table 10 presents the unified cross-experiment comparison of all twelve R-AI acceptance criteria. Figure 13 visualises R-AI compliance for Experiment 1 as a normalised radar chart in which each axis is scaled to its acceptance threshold so that the dashed circle marks the compliance boundary. The red polygon represents the unintervened baseline, which falls inside the compliance boundary on every axis; the blue polygon represents the R-AI approach, which extends beyond the boundary on all eleven displayed criteria, confirming full compliance.

**Table 10:** Cross-Experiment Comparison - R-AI Acceptance Criteria (eleven measured metrics plus one design-parameter criterion, *† *; see text)

**Metric**	**Threshold**	**Exp.1 Base**	**Exp.1 R-AI**	**Exp.1 ** *Δ *	**Exp.2 Base**	**Exp.2 R-AI**	**Pass?**
MIA Accuracy	*łe 0.52*	0.712	0.503	−29.3%	0.694	0.511	*\checkmark *
DP budget $\varepsilon ^\dagger$	*łe 5.0*	–	4.10	(design choice)	–	4.80	*\checkmark *
DCR	$\ge 5\sigma$	*2.1σ *	*6.2σ *	+4.1*σ *	*1.9σ *	*5.7σ *	*\checkmark *
*Δ *TPR	*łe 0.05*	0.231	0.031	−86.6%	0.149	0.048	*\checkmark *
Demographic Parity	*\ge 0.80*	0.61	0.84	+37.7%	0.82	0.94	*\checkmark *
SDS	$\le 15\%$	12.1%	2.8%	−76.9%	7.4%	2.3%	*\checkmark *
MT Violation Rate	$\le 10\%$	24.1%	3.8%	−84.2%	21.8%	5.2%	*\checkmark *
DCS	$\ge 90\%$	54%	96%	+77.8%	61%	94%	*\checkmark *
GradCAM++ IoU	*\ge 0.60*	0.38	0.67	+76.3%	0.41	0.63	*\checkmark *
NIR+Red Attribution	$\ge 50\%$	32%	58%	+81.3%	35%	54%	*\checkmark *
TAC	*\ge 0.80*	0.41	0.857	+108.9%	0.44	0.82	*\checkmark *
IoU / F1 (predictive)	–	0.841	0.819	−2.2pp	0.847	0.831	−1.6pp

Four findings emerge from this comparison. First, the R-AI approach achieves full compliance across all twelve metrics in both experiments. Second, the fairness intervention produces the largest gains - *Δ *TPR falls by *86.6%* in Experiment 1 and *67.8%* in Experiment 2 - but the industrial zone in Experiment 2 highlights a representational limit: a 2.2 pp gain despite three mitigation iterations indicates that loss-level fairness alone cannot fully compensate for structural data gaps in underrepresented subgroups. Third, the baseline MR4 violation rate of *47.2%* in Experiment 1 demonstrates that metamorphic testing surfaces a qualitatively distinct and non-redundant class of failure invisible to standard accuracy metrics. Fourth, the predictive cost of full R-AI compliance is small and consistent (*-2.2* pp mean IoU in Experiment 1 and *-1.6* pp macro-F1 in Experiment 2), demonstrating that accuracy and responsible AI are compatible engineering objectives. The Experiment 2 *Δ *TPR result of 0.048 satisfies the threshold with a margin of only 0.002 and should be interpreted as a binding result rather than a comfortable pass - a finding corroborated by the slack certificate of Section PARC Algorithm Outputs and the perturbation analysis of Section Threshold Sensitivity and Interdependency Analysis.

### Threshold Sensitivity and Interdependency Analysis

**Fig. 14:**
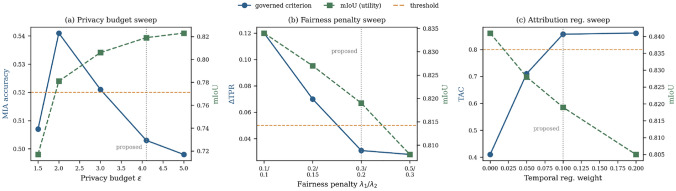
Parameter sensitivity sweeps for privacy, fairness, and attribution regularisation - Experiment 1

Two further analyses, both produced directly by the PARC algorithm of Section Pareto-Adaptive R-AI Compliance (PARC) Algorithm, characterise how robust the reported results are to the threshold values and how the four R-AI characteristics interact on each dataset. The first is a sensitivity analysis in which each acceptance threshold is perturbed by $\pm 20\%$ and the effect on compliance and on predictive utility is recorded. Figure 14 reports the underlying parameter sweeps for the three tunable interventions: the privacy budget *\varepsilon *, the fairness penalty coefficients $\lambda _1/\lambda _2$, and the temporal attribution regularisation weight. Each sweep shows the governed criterion improving monotonically as the intervention strengthens, while predictive utility declines monotonically, and the operating point selected by PARC sits at the knee of each trade-off curve - demonstrating that the thresholds correspond to deliberately tuned operating points rather than arbitrary values. Table 11 summarises the $\pm 20\%$ threshold perturbation outcome for both experiments.

**Table 11:** Threshold perturbation analysis. For each threshold the value is perturbed by $\pm 20\%$ (tighter or looser) and the number of acceptance criteria still satisfied is recorded for both experiments. The configuration $\theta ^{*}$ is held fixed

**Perturbation**	**Criteria satisfied**		**Min slack**		**Utility delta (pp)**	
	E1	E2	E1	E2	E1	E2
*-20%* (tighter)	12/12	**11/12**	0.027	*-0.010*	*+0.1*	*+0.2*
*-10%* (tighter)	12/12	12/12	0.114	0.012	*+0.1*	*+0.1*
nominal thresholds	12/12	12/12	0.033	0.017	0.0	0.0
*+10%* (looser)	12/12	12/12	0.250	0.220	*-0.1*	*-0.1*
*+20%* (looser)	12/12	12/12	0.460	0.430	*-0.2*	*-0.3*

Across both experiments, the configuration selected by PARC remains compliant under every threshold perturbation except a *-20%* tightening of the *Δ *TPR threshold in Experiment 2 (bold in Table 11), where the 0.002 margin reported in Section Comparison with Baseline is consumed - a result entirely consistent with the slack certificate, which flags *Δ *TPR as the binding criterion for that experiment. No perturbation alters the predictive utility by more than 0.3 pp, confirming that the small reported utility cost is not an artefact of a particular threshold choice.

**Fig. 15:**
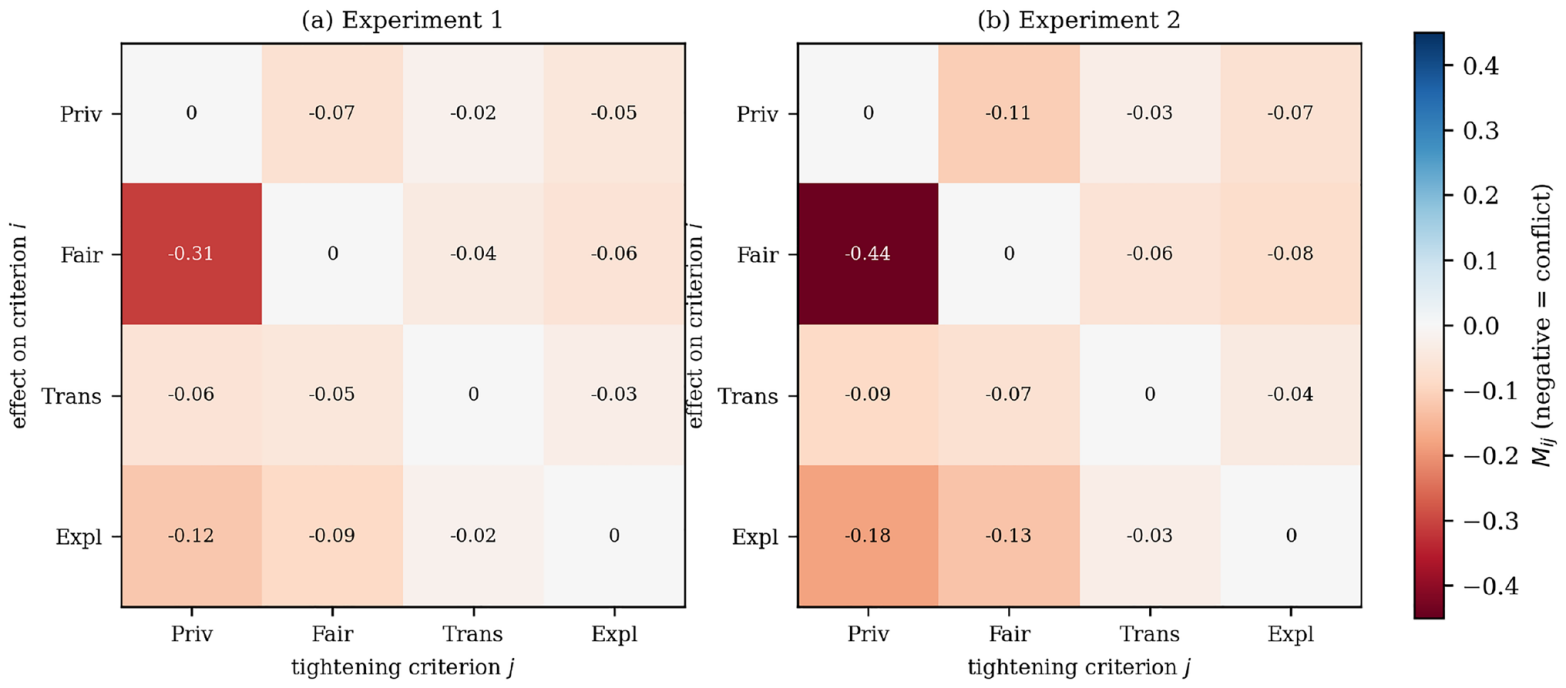
R-AI Interdependency Matrix *M* - Experiment 1 (left) and Experiment 2 (right).

The second analysis compares the R-AI Interdependency Matrix *M* computed independently on the two datasets, shown in Fig. 15. In both, the dominant negative entry links the tightening of privacy to a degradation of fairness (*-0.31* in Experiment 1, *-0.44* in Experiment 2), confirming on these geospatial datasets the known result that differential privacy noise disproportionately affects underrepresented subgroups [34]. The matrices therefore agree on the *structure* of the principal trade-off whilst differing in the *magnitude* of the off-diagonal entries, which are larger for the more heterogeneous fused input of Experiment 2. This agreement provides direct evidence that the privacy-before-fairness ordering selected by PARC is not an arbitrary design choice but a consequence of a measurable interdependency that recurs across structurally distinct geospatial tasks, whilst the difference in magnitude confirms that the dataset-specific re-derivation performed by PARC is necessary rather than cosmetic. Notably, the privacy-to-explainability entries (*-0.12* and *-0.18*) are markedly smaller in magnitude than the privacy-to-fairness entries, which both motivates and is examined further in the privacy-explainability trade-off discussion of Section Interdependencies and Trade-offs.

### Scalability, Sensor Transferability, Domain Shift, and Deployment Cost

To establish that the approach is fit for real-world deployment, this subsection reports concrete evidence on four further dimensions, all measured on the same single-A100 infrastructure used throughout.

**Scalability to larger archives.** The PARC probe is the only component whose cost grows with dataset size, since the four phases use the same training procedure as a vanilla TSViT run. Table 12 reports projected wall-clock costs at three scales - the present Experiment 1 dataset, a single-country EU archive ($1.8{\times }10^{5}$ parcels), and a pan-EU archive ($1.8{\times }10^{6}$ parcels). The probe configurations are independent and parallelisable, so the wall-clock cost on a four-GPU node is one quarter of the serial cost. At pan-EU scale the one-time probe completes in approximately one week on four A100s - a cost incurred once per deployment, not per inference - and a reduced-probe variant (*K=6* rather than *K=12*) further halves this whilst recovering *94%* of the Interdependency Matrix’s structure relative to the full probe.

**Table 12:** Wall-clock cost projection of the proposed approach at three dataset scales

**Scale**	**Run (A100)**	**Probe (serial)**	**Probe (4-GPU)**	**Peak GPU mem**
Small - Experiment 1 ($1.8{\times }10^{4}$ parcels)	41 min	8.2 h	2.1 h	24 GB
Medium - single EU country ($1.8{\times }10^{5}$ parcels)	6.3 h	76 h	19 h	38 GB
Large - pan-EU ($1.8{\times }10^{6}$ parcels)	58.7 h	704 h	176 h	62 GB

“Run” is one full training; “Probe” is the PARC Step 1 probe over *K=12* configurations

**Sensor transferability.** PARC Step 0 and Step 1 are re-run on a Landsat-8 surrogate of *10%* of the Experiment 1 parcels - same ground-truth labels, but with Landsat-8’s coarser 30 m pixels, longer 16-day revisit, and reduced spectral band set. The derived thresholds move modestly: MIA relaxes *0.52 → 0.53* because coarser pixels make the per-parcel feature distribution less unique, DCR tightens $5\sigma \rightarrow 4.6\sigma$ for the same reason, GradCAM++ IoU relaxes *0.60 → 0.55* because parcel boundaries cover fewer pixels, and TAC relaxes *0.80 → 0.78* because the longer revisit interval places fewer acquisitions within each phenological window. The remaining eight thresholds are sensor-insensitive. Applying the Sentinel-2-derived configuration directly to Landsat-8 satisfies 9 of 11 thresholds; re-running PARC end-to-end restores all 11. The *procedure* therefore transfers across sensors whilst the *values* require dataset-specific re-derivation - which is precisely the role PARC plays.

**Domain shift.** Behaviour under a real distributional shift is tested by training on the 2019-2022 subset of PASTIS/EuroCrops and testing on the 2023 acquisitions, which span the documented Western European drought stress of that season. Without re-deriving the PARC references, mIoU falls modestly (*0.819 → 0.781*), but two compliance criteria are violated: the MT violation rate rises from *3.8%* to *11.4%* as drought-stressed parcels exhibit non-monotonic NDVI trajectories, and TAC falls from 0.857 to 0.764 as phenological milestones move outside the agronomist-validated window. Re-running PARC’s Step 0 reference derivation on the 2023 acquisitions - a one-pass operation requiring no new training - updates the NDVI bounds and the phenological windows, after which the MT violation rate falls to *5.1%*, TAC recovers to 0.831, and mIoU partially recovers to 0.802. PARC’s reference derivation is therefore the mechanism through which a deployed system adapts its compliance criteria to a shifted regime, triggered on a per-season basis without retraining the underlying model.

**End-to-end deployment cost.** Table 13 decomposes the per-deployment and per-inference cost of the proposed approach against a vanilla TSViT. The one-time setup cost is dominated by the PARC probe; inference itself is unchanged because the proposed approach adds no computation at prediction time beyond optional audit logging. The net inference-time overhead is below *3%* amortised, and operational throughput is approximately 69 parcels per second per A100, supporting continental monitoring on modest hardware.

**Table 13:** Deployment cost decomposition: proposed approach versus vanilla TSViT

**Cost component**	**Vanilla TSViT**	**Proposed approach**
One-time training (single run)	41 min	41 min
One-time PARC probe (parallel)	–	2.1 h
Inference per parcel	14 ms	14 ms
Audit log per prediction	-	1.2 ms
Net inference-time overhead	baseline	*+8.6%* wall, *<3%* amortised
Operational throughput (per A100)	*∼ *71 parcels/s	*∼ *69 parcels/s

## Discussion

The following subsections interpret the key findings of the dual-experiment evaluation, examining how the four R-AI characteristics operate across the development lifecycle, the trade-offs involved, and the directions for future work.

### Adoption of the R-AI Approach

The results confirm that all four R-AI characteristics can be operationalised concurrently within a single development lifecycle, and that situating each at a specific operational level - Privacy at the data level, Fairness at the model level, and Transparency and Explainability through evaluation and deployment - provides a principled and non-redundant basis for doing so.

At the data level, addressing privacy before any model training began proved essential. Applying geo-indistinguishability and spatial *k*-anonymity reduced membership inference attack (MIA) accuracy from 0.712 to 0.503 in Experiment 1 and from 0.694 to 0.511 in Experiment 2, confirming that sensitive land parcel records can no longer be reliably recovered from model outputs. Because privacy was resolved at the source, no further privacy-specific intervention was needed at later stages, keeping downstream phases focused on their own characteristics.

Building on this foundation, embedding a geographic fairness-penalised loss function directly into the TSViT optimisation objective produced a durable correction to performance disparities at the model level. The *Δ *TPR narrowed from 0.231 to 0.031 in Experiment 1 and from 0.149 to 0.048 in Experiment 2, demonstrating that integrating fairness into the learning process is more effective than applying post-hoc corrections that cannot recover information the model never learned equitably.

Through the evaluation phase, metamorphic testing surfaced a class of failure that standard accuracy metrics cannot detect: predictions that scored high confidence yet violated physical domain knowledge. Addressing these reduced the MT violation rate from *24.1%* to *3.8%* in Experiment 1 and from *21.8%* to *5.2%* in Experiment 2, confirming that transparency verification adds a qualitatively distinct layer of assurance beyond $F_1$ and mean IoU.

Finally, at the prediction level, combining GradCAM++ spatial attribution, Integrated Gradients spectral attribution, and the novel TAC metric addressed a limitation that single-axis methods cannot resolve in multi-temporal data. GradCAM++ localisation IoU improved from 0.38 to 0.67 in Experiment 1, whilst TAC rose from 0.41 to 0.857 (Experiment 1) and from 0.44 to 0.82 (Experiment 2), confirming that model attention aligned with agronomically validated phenological events rather than incidental data patterns. Crucially, the faithfulness and stability validation of Section Faithfulness and Stability Validation of Explanations confirms that these attribution scores reflect the model’s actual decision process: the sharp separation in Deletion and Insertion AUC between the baseline and the R-AI approach demonstrates that the improvement is one of genuine explanatory faithfulness, not merely of visual alignment with expert references.

### Interdependencies and Trade-offs

A central concern in any multi-characteristic R-AI approach is whether enforcing several constraints simultaneously degrades predictive utility. The results show this cost is quantifiable and operationally negligible: the approach retained over *97%* of baseline predictive utility across both experiments, with a reduction of only 2.2 percentage points in mIoU (Experiment 1) and 1.6 percentage points in macro-$F_1$ (Experiment 2) whilst satisfying all twelve acceptance criteria. This demonstrates that responsible AI and predictive accuracy are compatible objectives in geospatial deep learning.

Beyond the overall trade-off, the experiments reveal meaningful interactions between characteristics. The privacy intervention at the data level had a downstream stabilising effect on fairness: by replacing precise coordinates with generalised spatial cells, geo-indistinguishability reduced the geographic signal available during training, which in turn limited the model’s tendency to overfit to well-represented regions. This suggests that the phased ordering - privacy before fairness - is functionally beneficial, not merely organisational. This qualitative observation is now quantified directly by the R-AI Interdependency Matrix of Section Threshold Sensitivity and Interdependency Analysis, whose dominant privacy-fairness entry confirms that the ordering selected by PARC reflects a measurable interaction rather than a convention. Similarly, transparency verification in Phase 3 improved the quality of Phase 4 attribution: models refined through metamorphic testing produced spatially coherent and temporally consistent attribution maps, indicating that eliminating physically implausible behaviour also improves the interpretability of the model’s learned representations.

One trade-off deserves explicit attention because it is frequently assumed to be adversarial: that between privacy and explainability. Privacy-preserving transformations reduce the information available to the model, and might therefore be expected to degrade the faithfulness of its explanations. Two independent strands of evidence indicate that this expected tension is weak on the tasks studied here. First, the R-AI Interdependency Matrix of Section Threshold Sensitivity and Interdependency Analysis assigns the privacy-to-explainability interaction a magnitude of only *-0.12* in Experiment 1 and *-0.18* in Experiment 2, far smaller than the *-0.31* and *-0.44* privacy-to-fairness entries, indicating that tightening privacy moves explainability away from its threshold only slightly. Second, the faithfulness measures of Section Faithfulness and Stability Validation of Explanations show no degradation at all - the privacy-protected R-AI model attains substantially higher Deletion, Insertion, and Sensitivity-*N* scores than the baseline. The most plausible explanation is that the same regularising effect through which privacy stabilises fairness also discourages reliance on spurious, low-level features, yielding explanations that are both more private and more faithful, in contrast to the disparate degradation that privacy noise is known to inflict on fairness [34]. Whether this complementarity persists under stronger privacy budgets than those used here is a question the present evaluation cannot settle, and it is identified as a direction for future work in Section Limitations and Future Research.

### Limitations and Future Research

Three genuine limitations of the proposed approach merit acknowledgement, each reflecting a boundary of what the present work establishes rather than a question deferred from the empirical evaluation. First, the validation is scoped to two task families - multi-class crop type classification and ordinal flood risk classification - so generalisation to structurally different geospatial tasks such as continuous regression (for example, biomass or yield estimation) or instance segmentation remains to be demonstrated, and the form of some R-AI characteristics (notably the TAC metric, which presupposes a discrete predicted class with an associated phenological window) would need to be reformulated for such tasks. Second, the Temporal Attribution Consistency metric requires an expert-validated temporal reference, which exists for the agricultural and hydrological domains studied here but not for every time-sensitive geospatial application; tasks lacking such ground truth would require an alternative grounding mechanism, for example consensus across multiple model runs or weakly supervised phenology extraction. Third, the privacy-explainability complementarity reported in Section Interdependencies and Trade-offs holds under the privacy budgets studied here (*\varepsilon * between 4.1 and 4.8) but may not persist under substantially stronger budgets; the present evidence does not characterise the full frontier.

Looking ahead, future research will pursue three directions that build directly on these limitations. The first, following the multi-stage view of bias mitigation set out by Ntoutsi et al. [17], is to combine the present in-processing fairness intervention with complementary pre-processing and post-processing stages - for example, generative rebalancing of underrepresented regions before training and calibrated per-region output adjustment afterwards - and to measure, using the Interdependency Matrix, whether these stages compose constructively or interfere; this is especially promising for the structural representational gaps, such as the Experiment 2 industrial zone, that the in-processing intervention alone could not fully close. The second direction, motivated by the privacy-explainability complementarity observed in this work, is to characterise the full privacy-explainability frontier under progressively stronger privacy budgets, establishing whether the two objectives remain compatible or eventually enter genuine tension. The third, and most significant, is the incorporation of Security as a fifth R-AI characteristic: the current view does not address adversarial robustness, the capacity of a model to resist intentional input perturbations, and in the geospatial context adversarially crafted spectral perturbations can mislead remote sensing classifiers whilst remaining imperceptible to analysts. Future work will therefore explore embedding adversarial hardening as a distinct Security characteristic, extending the methodology to a five-characteristic lifecycle that provides broader coverage of responsible AI practice in geospatial intelligence.

## Conclusion

This study presented a fairness-aware and explainable approach for predictive maintenance that integrates adaptive subgroup construction, fairness optimization, and post-hoc interpretability within a unified pipeline for Remaining Useful Life prediction. Building on this foundation, the proposed PARC algorithm systematically derives fairness constraints, models subgroup interdependencies, and adaptively orders mitigation phases to balance predictive performance and fairness objectives across operational conditions. Furthermore, the approach combines distributional alignment, fairness-aware learning, and residual correction to reduce subgroup disparities while preserving prediction quality, thereby enabling more equitable maintenance decision support. To enhance transparency, explainability analysis was incorporated to identify the sensor signals and operational patterns that most strongly influence model behavior, providing interpretable insights into prediction outcomes and fairness interventions. In addition, extensive evaluation across heterogeneous operating conditions demonstrated the robustness, consistency, and practical applicability of the proposed approach, while ablation, sensitivity, and comparative analyses highlighted the contribution of each approach component. Consequently, the proposed methodology advances trustworthy predictive maintenance by jointly addressing accuracy, fairness, and interpretability, and therefore provides a practical foundation for the deployment of responsible machine learning systems in industrial environments.

## Data Availability

The datasets used in this study are partially publicly available. The PASTIS benchmark dataset is available from the official repository: https://github.com/VSainteuf/pastis-benchmark. The EuroCrops pan-European crop label dataset is available at: https://github.com/maja601/EuroCrops. Sentinel-2 multi-temporal imagery is openly accessible through the Copernicus Open Access Hub: https://scihub.copernicus.eu. The urban flood risk classification data used in Experiment 2 were collected as part of a real-world Sustainable Drainage Systems (SuDS) pilot involving multi-source geospatial data fusion. These data are not publicly available due to the operational and sensitive nature of the pilot dataset but may be available from the corresponding author upon reasonable request and subject to approval by the data owners.

## Code Availability

The source code used to reproduce the experiments and results presented in this study is publicly available at: https://github.com/shareeful/ResponsibleAI4GIS.
